# Proline Homologation in Melanostatin Neuropeptide: Discovery of Potent Modulators of the Dopamine D_2_ Receptors

**DOI:** 10.1021/acsmedchemlett.5c00287

**Published:** 2025-07-24

**Authors:** Ivo E. Sampaio-Dias, Hugo F. Costa-Almeida, Xavier C. Correia, Sara C. Silva-Reis, Vera M. Costa, José Brea, María I. Loza, José E. Rodríguez-Borges, Xerardo García-Mera

**Affiliations:** † LAQV/REQUIMTE, Department of Chemistry and Biochemistry, Faculty of Sciences, 131674 University of Porto, 4169-007 Porto, Portugal; ‡ UCIBIO − Applied Molecular Biosciences Unit, Laboratory of Toxicology, Department of Biological Sciences, Faculty of Pharmacy, 131674 University of Porto, 4050-313 Porto, Portugal; § Associate Laboratory i4HB − Institute for Health and Bioeconomy, Laboratory of Toxicology, Department of Biological Sciences, Faculty of Pharmacy, 131674 University of Porto, 4050-313 Porto, Portugal; ∥ Innopharma Screening Platform. Biofarma Research Group. Centre of Research in Molecular Medicine and Chronic Diseases (CIMUS), University of Santiago de Compostela, E-15782 Santiago de Compostela, Spain; ⊥ Department of Organic Chemistry, Faculty of Pharmacy, University of Santiago de Compostela, E-15782 Santiago de Compostela, Spain

**Keywords:** Dopamine D_2_ Receptors, Melanostatin, Parkinson’s Disease, Pipecolic Acid, Positive Allosteric Modulators

## Abstract

Melanostatin (MIF-1) is a naturally occurring neuropeptide acting as a positive allosteric modulator (PAM) of dopamine D_2_ receptors (D_2_R), underscoring its potential for therapeutic use in central nervous system disorders associated with dopaminergic dysregulation, including depression, drug addiction, restless legs syndrome, tardive dyskinesia, and Parkinson’s disease. In this work, a new series of MIF-1 analogs using l-pipecolic acid as an l-proline surrogate was synthesized and pharmacologically evaluated by functional assays at the D_2_R. In this series, methyl l-pipecolyl-l-leucylglycinate (**9**) was found to exhibit superior performance compared to MIF-1 by promoting a 4.1- and 4.2-fold increase of dopamine potency at 0.01 and 1 nM, respectively. *In silico* conformational studies demonstrate that **9** preferentially adopts a γ-turn, corroborating that neither the C-terminal carboxamide nor the postulated type II β-turn conformation is required for PAM activity. Toxicological assays in human dopaminergic SH-SY5Y neuronal cells show that this compound exhibits no significant toxicity up to 100 μM in the MTT reduction and neutral red uptake assays.

## Graphical abstract

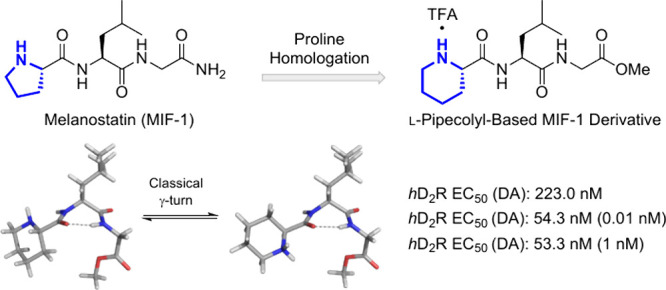

Melanostatin, also referred to as melanocyte-stimulating hormone release-inhibiting factor-1 (MIF-1, Figure 1), is an endogenous hypothalamic neuropeptide, formally known as l-prolyl-l-leucylglycinamide, that exhibits biological activity within the central nervous system (CNS).^1–9^ MIF-1 was first isolated from hypothalamic bovine extracts in 1971;^10^ however, its biosynthesis remains unclear. Studies indicate that MIF-1 can be produced upon exocyclic cleavage of the oxytocin hormone,^11^ while others demonstrate it can be obtained from l-tyrosyl-l-prolyl-l-leucylglycinamide (Tyr-MIF-1) neuropeptide.^12^ In the brain, MIF-1 blocks the effects of opioid receptor activation,^1–3^ inhibits the release of other neuropeptides such as α-melanocyte-stimulating hormone,^4^ and also potentiates melatonin activity.^5^ Among the vast repertoire of biological activities associated with MIF-1, its interaction with the dopamine receptors is one of the most extensively explored due to its potential applications in dopamine-related disorders of the CNS. Experiments using radiolabeled ligands have demonstrated that MIF-1 increases the specific binding of dopamine receptor agonists such as *N*-propylapomorphine and quinpirole to dopamine receptor subtypes D_2L_, D_2S_, and D_4_ in a dose-dependent fashion, producing typical concentration–response curves exhibiting a bell or “inverted U” shape.^13^ Interestingly, MIF-1 has no meaningful influence on the binding of agonists to D_1_ and D_3_ receptors or antagonists to the D_2L_ receptors or other G protein-coupled receptors, thus exhibiting D_2_-specificity.^14,15^ This feature makes MIF-1 the only known endogenous positive allosteric modulator (PAM) selectively targeting the D_2_ receptors (D_2_R).^16^ So far, the exact allosteric binding site for MIF-1 has not yet been identified.^17^ Structural analysis by X-ray crystallography^18^ and ^1^H nuclear magnetic resonance (NMR) experiments in deuterated dimethyl sulfoxide (DMSO-*d*_6_) hint that MIF-1 acquires a type II β-turn conformation (Figure 1).^19^ However, laser Raman experiments of MIF-1 in D_2_O indicate that MIF-1 might adopt different conformations in aqueous solution from those observed in the solid state and DMSO-*d*_6_.^19^ The existence of other bioactive conformations is further supported by structure–activity relationship studies with MIF-1 derivatives reported by our group^20–23^ and others.^24–31^

**Figure 1:**
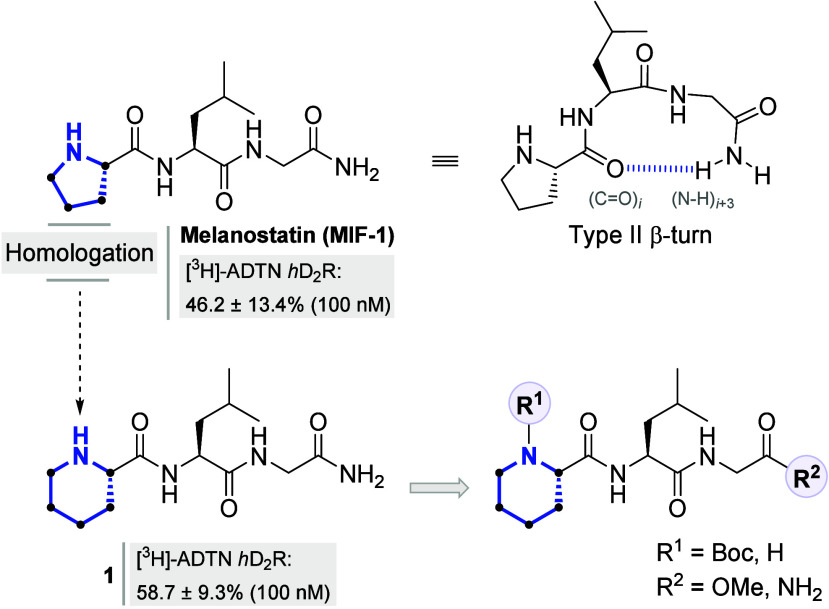
Structure of melanostatin (MIF-1), including its putative bioactive type II β-turn conformation, and the structures of its homologue **1** and the novel l-pipecolyl-based MIF-1 derivatives described in this work. The blue dashed line represents the intramolecular hydrogen bond responsible for the type II β-turn conformation.

The notable PAM activity and D_2_-selectivity of MIF-1 make it a promising lead compound for the treatment of several CNS-related disorders associated with D_2_R dysregulation, such as depression,^6^ drug addiction,^32^ tardive dyskinesia,^7^ restless legs syndrome,^8^ and Parkinson’s disease.^9^

In a landmark study, Johnson and co-workers explored the influence of the l-proline residue on the modulatory activity of the dopamine receptors isolated from bovine caudate tissue.^33^ To this end, the l-proline residue was replaced with several heterocyclic amino acids. Binding assays showed that replacement of l-proline with l-pipecolic acid in the MIF-1 structure, namely, the l-pipecolyl-l-leucylglycinamide analog (**1**, Figure 1), exhibited a statistical (*p* < 0.05) enhancement in the specific binding of radiolabeled 2-amino-6,7-dihydroxy-1,2,3,4-tetrahydronaphthalene (tritiated, [^3^H]-ADTN) to dopamine receptors in a dose-dependent manner. The maximal effect was observed at 100 nM, with a specific binding of 58.7 ± 9.3%,^33^ compared to 46.2 ± 13.4% for the parent neuropeptide at the same concentration.^33^ Despite these interesting results, to date, no further validation of the PAM activity has been provided in the literature by functional assays for MIF-1 derivative **1**.

In this work, in addition to **1**, novel structure-related derivatives were synthesized and pharmacologically evaluated by functional assays on dopamine D_2_R, including a study of their toxicological profiles on differentiated SH-SY5Y cells.

**Organic Synthesis**.

The synthesis route for the preparation of pipecolyl-based MIF-1 derivatives is outlined in Scheme 1.

**Scheme 1:**
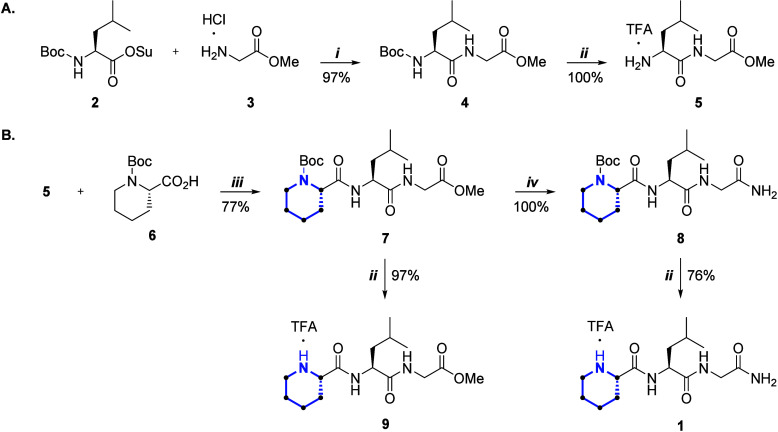
Synthesis of Dipeptide **5** (A) and the Pipecolyl-Based MIF-1 Derivatives **1** and **7–9** (B). *Reagents and Conditions*: i. Et_3_N, Anhydrous CH_2_Cl_2_; ii. TFA, Anhydrous CH_2_Cl_2_, iii. Et_3_N, TBTU, Anhydrous CH_2_Cl_2_; iv. 7 M NH_3_ in Methanol

Following a convergent synthesis approach, the C-terminal dipeptide was first synthesized starting with the peptide coupling of preactivated (succinimidyl ester) leucine derivative **2** with methyl glycinate hydrochloride (**3**) in the presence of triethylamine (Et_3_N), affording dipeptide **4** in 97% yield (Scheme 1A). Then, the Boc group was removed by acidolysis using trifluoroacetic acid (TFA), yielding dipeptide **5** in quantitative yield as an ammonium trifluoroacetate salt (Scheme 1A).^22,34^

With dipeptide **5** in hand, tripeptide **7** was obtained by peptide coupling with Boc-l-pipecolic acid (**6**), under mild conditions. This process involves carboxylic acid activation with 2-(1*H*-benzotriazol-1-yl)-1,1,3,3-tetramethyluronium tetrafluoroborate (TBTU) under basic conditions using Et_3_N (Scheme 1B). Following this protocol, tripeptide **7** was obtained with a 77% yield (Scheme 1B). Then, the methyl ester of **7** was converted into the corresponding primary amide **8** by ammonolysis using a commercial solution of 7 M NH_3_ in methanol, affording the product in a quantitative yield (Scheme 1B). The last step of the synthesis route was the removal of the Boc group from tripeptides **7** and **8** by acidolysis using TFA, delivering tripeptides **9** and **1** in 97 and 76% yields, respectively, as ammonium trifluoroacetate salts (Scheme 1B). The lower yield of compound **1** is attributed to product loss during chromatographic purification. The structural elucidation of tripeptides **1** and **7–9** was performed by 1D (^1^H, ^13^C­{^1^H}, and DEPT-135) and 2D (COSY and HSQC) NMR and high-resolution electrospray ionization mass spectrometry (ESI-HRMS). The data obtained are in perfect agreement with the chemical structures of the target compounds (Figures S1–S24).

**Dopamine D_2_ Receptor Functional Assays**.

Pharmacological functional assays were conducted at human D_2_R expressed in Chinese hamster ovary (CHO) cells to characterize the PAM activity of the target compounds **1** and **7–9**, namely, the half-maximal effective concentration (EC_50_) and maximal efficacy (*E*_max_) of dopamine through measurement of cyclic adenosine monophosphate (cAMP) levels following D_2_R activation, using homogeneous time-resolved fluorescence (HTRF) technology.^20,21,35^

Dopamine concentration–response curves were recorded in the presence and absence of **1** and **7–9**, using MIF-1 as a positive control. The resulting data was normalized to the maximal dopamine response (*E*_max_) as previously described by our research group.^20,21,35^ The corresponding dopamine concentration–response curves are depicted in Figure 2.

**Figure 2:**
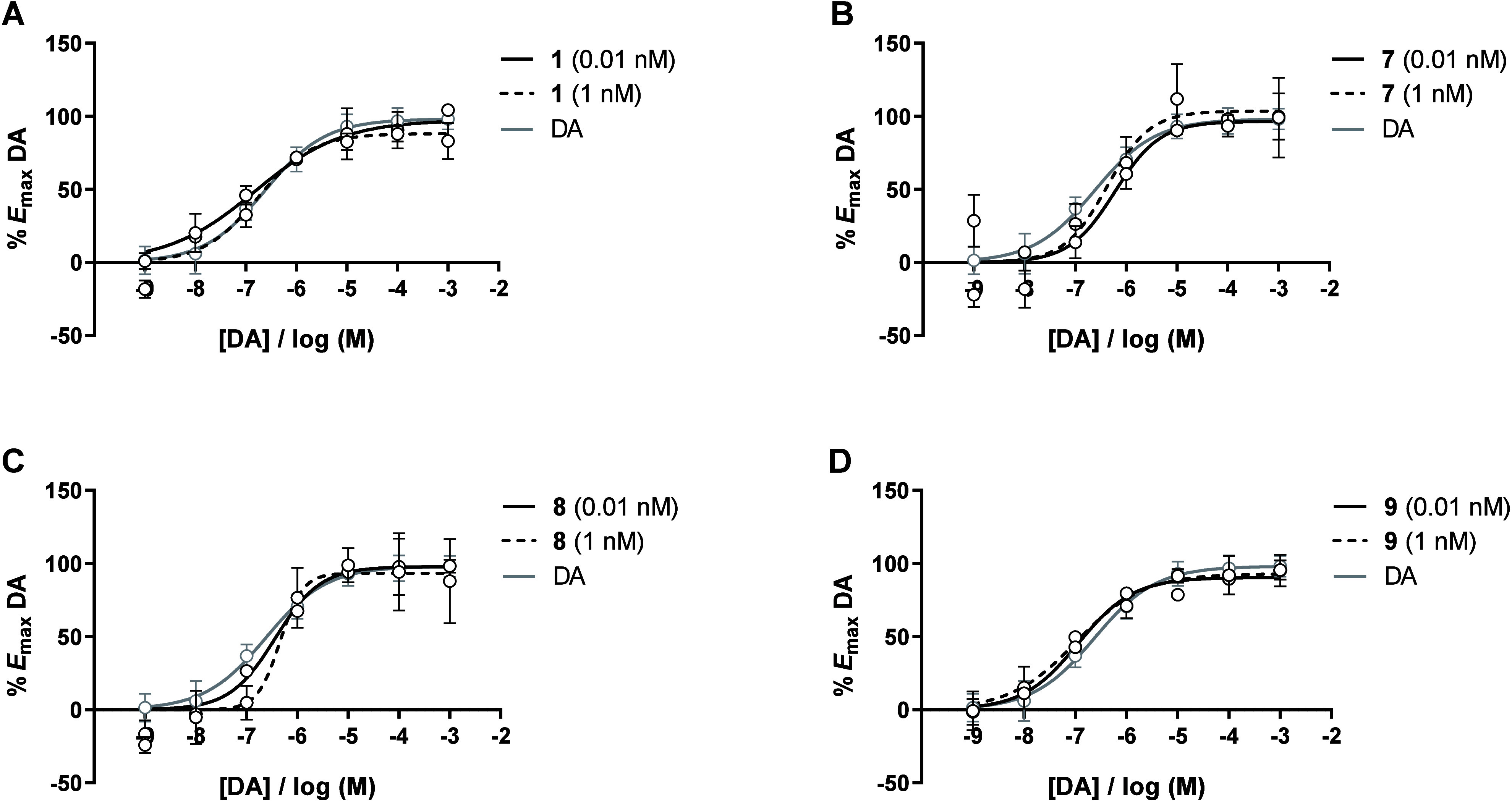
Concentration–response curves of dopamine (DA) in the presence of 0.01 nM (solid black line) and 1 nM (dashed black line) of compounds **1** (A), **7** (B), **8** (C), and **9** (D). The concentration–response curve of DA alone is included in all graphics for comparative purposes (gray solid line). Data points represent the mean ± standard deviation of two independent experiments with duplicate measurements.

The EC_50_ obtained for dopamine alone was 223.00 nM. At 0.01 nM, MIF-1 derivatives **1** and **9** (Figures 2A and 2D, respectively) induced a left-shift of the dopamine concentration–response curves, demonstrating a 1.8- and 4.1-fold increase in dopamine potency, respectively (EC_50_ = 120.66 nM for **1** and EC_50_ = 54.29 nM for **9**), without impact on the maximal response (*E*_max_ = 100% for **1** and **9**). Conversely, MIF-1 exhibited a 3.8-fold increase in dopamine potency at 0.01 nM (EC_50_ = 59.05 nM, Figure S25). At this concentration, MIF-1 and derivative **9** exhibit similar PAM activity (*p* > 0.05).

Interestingly, at 1 nM, **9** was able to retain the PAM activity by promoting a 4.2-fold increase in dopamine potency (EC_50_ = 53.28 nM, Figure 2D), while the parent neuropeptide exhibited a less pronounced reduction of the EC_50_ of dopamine (EC_50_ = 72.41 nM, Figure S25) with no effect on the efficacy (*E*_max_ = 100%). At this concentration (1 nM), compound **1** was found to be devoid of PAM activity, as it did not alter the EC_50_ of dopamine.

Altogether, in this series of l-pipecolyl-based MIF-1 derivatives, compound **1** was found to be less potent than MIF-1 in the functional assays at both concentrations tested (0.01 and 1 nM), while compound **9** was able to retain (0.01 nM) or even outperform (1 nM) the PAM activity of the MIF-1 neuropeptide. The remaining compounds, **7** and **8**, did not improve the EC_50_ of dopamine at any of the concentrations tested (Figures 2B and 2C, respectively).

To exclude intrinsic agonist activity and validate the PAM mechanism of the best-performing MIF-1 derivatives **1** and **9**, the same experiments were conducted in the absence of dopamine and compared to the concentration–response curve of dopamine alone (Figure 3).

**Figure 3:**
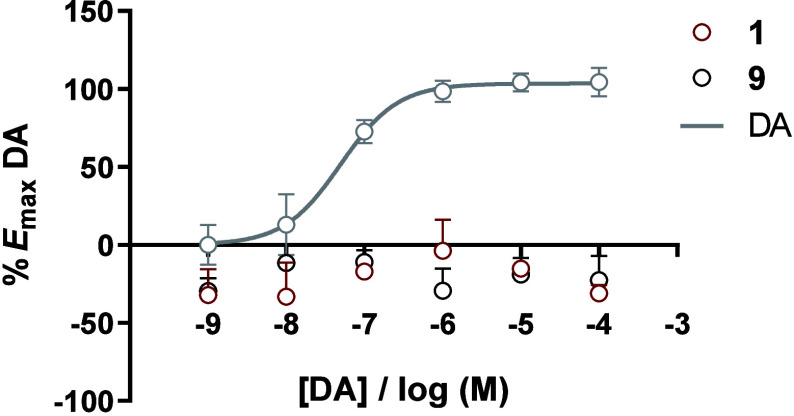
Concentration–response for compounds **1** and **9** obtained at human D_2_R in the absence of dopamine (DA), with the DA concentration–response curve included for reference. Data are presented as the mean ± standard deviation from three independent experiments.

The data presented in Figure 3 demonstrate that both **1** and **9** display no intrinsic agonistic effect and exclude the possibility that these compounds behave as agonists, promiscuous allosteric agonists, or ago-allosteric modulators, thus corroborating the PAM mechanism for these compounds.

Collectively, the results obtained from the functional assays support the conclusion that compounds **1**, **9**, and MIF-1 share the same underlying mechanism of action. Interestingly, among this series of MIF-1 derivatives, the presence of the carbamate group in derivatives **7** and **8** was found to reduce (**7**) or even abolish (**8**) the PAM activity. Moreover, the replacement of the primary amide (**1**) by the methyl ester group (**9**) resulted in higher PAM activity. Prior studies from our research group have shown that, although the C-terminal primary amide of MIF-1 is deemed a key pharmacophore, it is not essential for its PAM activity.^20–22,36^

The acceptance of the amide-to-ester replacement in the MIF-1 neuropeptide provides valuable insights into the pharmacology of MIF-1. While this isosteric substitution maintains a planar geometry,^37^ replacing the amide NH group (H-bond donor) with an ester oxygen (H-bond acceptor) and the amide carbonyl (strong H-bond acceptor) with an ester carbonyl (weaker H-bond acceptor) is known to impact folding energetics.^37,38^

The results obtained show that, while in the binding assays, compound **1** was reported to enhance the specific binding of [^3^H]-ADTN to the dopamine receptors at 10 and 100 nM,^33^ in the functional assays, **1** is able to improve the dopamine potency at the D_2_R at 0.01 nM, albeit achieving only half of the dopamine potency in the presence of MIF-1 at this concentration. However, unlike MIF-1, **1** was found to be devoid of PAM activity at 1 nM. Our results suggest that the additional methylene group of l-pipecolic acid (**1**), in contrast with l-proline (MIF-1), seems to benefit from the concomitant presence of the ester function (**9**). This can be attributed to the overall lipophilicity and additional enthalpic binding contributions, promoting either better fitting or specificity to the D_2_R allosteric binding site.

It should be noted, however, that functional assays do not provide information about the binding site(s), so the possibility of these compounds exploring different allosteric binding sites at the D_2_R cannot be discarded.

***In Silico*** **Studies**.

Gas-phase energy minimization calculations were performed with Gaussian 09 to examine the preferential conformations of compounds **1** and **9**, aiming to obtain additional structure–activity insights.^39^ Computations were performed at the PBE0/6–31G­(d,p) level of theory in implicit water using the integral equation formalism polarizable continuum model (IEFPCM) as the solvation model. Both neutral and protonated forms of **1** and **9** were considered, with the protonated species reflecting the likely predominant state at physiological pH. The optimized geometries for **1** and **9** are shown in Figure 4 (and Tables S1–S4).

**Figure 4:**
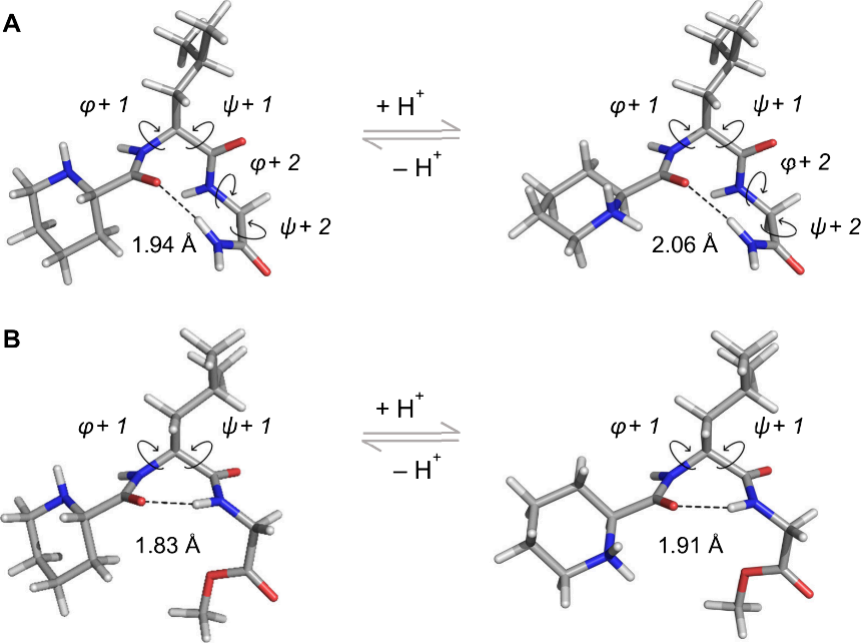
Optimized geometries of **1** (**A**) and **9** (**B**) at the PBE0/6–31G­(d,p) level of theory in implicit water using the IEFPCM solvation model, exhibiting a type I′ β-turn and a classical γ-turn, respectively, assisted by intramolecular hydrogen bonds (dashed black lines).

Energy minimization studies show that both neutral and protonated species of **1** preferentially adopt type I′ β-turns (Figure 4A), while **9** forms γ-turn conformations (Figure 4B), as detailed in the Supporting Information. It is important to note that these minimized structures represent low-energy conformations in an implicit solvent and do not necessarily reflect the receptor-bound active conformations. Given the intrinsic conformational flexibility of these small molecules, alternative conformations may coexist at equilibrium.

Interestingly, Aizpurua and co-workers reported MIF-1 peptidomimetics bearing a β-lactam motif as an l-leucine surrogate that can equilibrate between type II β-turn and γ-turn conformations.^40^ In the present series, however, compound **9** is unable to adopt β-turn conformations due to the absence of a C-terminal carboxamide group (Figure 4B). β-Turn formation generally requires a stabilizing intramolecular hydrogen bond between the carbonyl oxygen of the amino acid residue at position *i* and the amide hydrogen of the residue at position *i*+3. Since compound **9** lacks this key hydrogen bond donor, it cannot engage in β-turn conformations (*cf*. Figures 4A and 4B).

Collectively, these observations provide useful insights into MIF-1 structure–activity relationships. While both **1** and **9** show potent PAM activity at the D_2_R, only compound **1** retains the structural features compatible with β-turn formation. Notably, compound **9**, the most potent compound in this series, lacks the C-terminal carboxamide group, which is a structural requirement to adopt β-turn conformations. These findings challenge prior assumptions and, at the same time, support emerging evidence of flexible pharmacophore requirements^20–22^ and alternative bioactive conformations in MIF-1 derivatives beyond the canonical type II β-turn.^20–30^

**Cytotoxicity Assays in Differentiated SH-SY5Y Cells**.

Human-derived SH-SY5Y neuroblastoma cells are widely used in cellular-based models of neurodegenerative diseases of the CNS.^20,21,35^ ^,^ ^41–43^ To determine the toxicological profiles of the pharmacologically active MIF-1 derivatives (compounds **1** and **9**), a 6-day cellular differentiation protocol using retinoic acid (RA) and 12-*O*-tetradecanoylphorbol-13-acetate (TPA) was implemented to induce SH-SY5Y cells to acquire features of human dopaminergic neurons, including the increased expression of dopamine transporters and tyrosine hydroxylase, a key enzyme in catecholamine synthesis,^44^ being thus relevant as an *in vitro* model of Parkinson’s disease.^42,43^ This differentiated model also exhibits a lower mitotic rate and increased resistance to dopamine neurotoxicity.^45^

Following a protocol established in our research group,^21,35,41,46^ these compounds were tested at 100 μM concentration using the 3-(4,5-dimethylthiazol-2-yl)-2,5-diphenyltetrazolium bromide (MTT) reduction and the neutral red (NR) uptake assays. The results obtained are shown in Figure 5.

**Figure 5:**
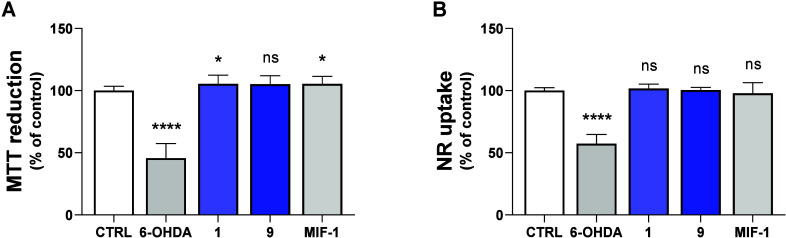
Neurotoxic effects of the synthesized compounds were examined via MTT reduction (A) and NR uptake (B) assays in differentiated SH-SY5Y neuronal cells. Cells were incubated for 48 h with **1**, **9**, MIF-1 (100 μM), or 6-OHDA (125 μM). Compounds were solubilized in phosphate-buffered saline (PBS) solution. Results are reported as a percentage relative to the control (PBS) and shown as mean ± standard deviation. Data were collected from 16 to 36 wells across 9 independent experiments for the MTT reduction assay and from 12 wells and 3 independent experiments for the NR uptake assay. Statistical analyses were performed using the analysis of variance (ANOVA) test, followed by Tukey’s *post hoc* test (*p < 0.05 and ****p < 0.0001 vs control).

In both assays, 6-hydroxydopamine (6-OHDA), a hydroxylated derivative of dopamine commonly employed as a neurotoxicant to mimic the phenotype of Parkinson’s disease in several *in vitro* and *in vivo* models,^47–49^ was used as a positive control for cytotoxicity (125 μM, Figures 5A and 5B). In the MTT reduction assay (Figure 5A), the 6-OHDA condition led to pronounced neurotoxicity (45.64 ± 11.85%, *p* < 0.0001). In contrast, neither MIF-1 (105.49 ± 6.00%), **1** (105.39 ± 7.10%), nor **9** (105.17 ± 6.80%) caused a significant cytotoxic effect at 100 μM in the MTT reduction assay (Figure 5A). In fact, there was a small increase in the mitochondrial reduction ability under MIF-1 and **1** conditions.

In the NR uptake assay (Figure 5B), the 6-OHDA condition led to pronounced neurotoxicity (57.26 ± 7.41%, *p* < 0.0001), while the MIF-1 (97.77 ± 8.56%), **1** (101.66 ± 3.48%), and **9** (100.49 ± 2.09%) conditions did not exhibit a significant cytotoxic effect up to 100 μM.

In addition to the potent PAM activity at subnanomolar concentration, these results indicate that MIF-1 derivatives **1** and **9** are devoid of significant cytotoxicity toward human-derived differentiated SH-SY5Y neuroblastoma cells up to 100 μM, exhibiting toxicological profiles comparable to that of MIF-1 neuropeptide.

In conclusion, the homologation strategy of l-proline in MIF-1 neuropeptide was found to be feasible, leading to the discovery of two l-pipecolyl-based MIF-1 derivatives (**1** and **9**) exhibiting potent PAM activity at the D_2_R. MIF-1 derivative **1** improves the potency of dopamine at 0.01 nM, albeit achieving only half of the dopamine potency when compared with that of MIF-1. Interestingly, while the pyrrolidine ring system of MIF-1 is not deemed as an essential structural motif to elicit PAM activity (**1** vs MIF-1), the results suggest that the PAM activity of l-pipecolyl-based MIF-1 derivatives can be improved and properly balanced with isosteric modifications at the C-terminal position (**1** vs. **9**). In this series, ester **9** exhibited a similar (0.01 nM) or improved (1 nM) PAM activity compared to the parent neuropeptide, while **1** was found inactive at the highest concentration tested.

Importantly, the PAM activity observed for **9** cannot be attributed only to increased overall lipophilicity, since its carbamate precursor (**7**) was found inactive. It is noteworthy to mention that while the *in silico* studies showed that **1** preferentially adopts a type I′ β-turn conformation, it does not exclude the possibility of this peptidomimetic acquiring the putative type II β-turn as the bioactive conformation. Interestingly, the amide-to-ester chemical modification prevents compound **9** from adopting β-turn conformations, suggesting a preference for a classical γ-turn in aqueous media. These results open new avenues for exploring noncanonical pharmacophores in MIF-1 drug design.

Altogether, the structure–activity relationship insights provided in this work, combined with the potent PAM activity at the D_2_R and the favorable toxicological profiles of the pipecolyl-based MIF-1 derivatives, are expected to guide the rational design and development of novel anti-Parkinson’s drugs.

**Safety Statement**.

No unexpected or unusually high safety hazards were encountered.

## Supplementary Material



## Notes

Detailed synthetic procedures and copies of 1D (^1^H, ^13^C­{^1^H}, DEPT-135) and 2D (COSY, HSQC) NMR spectra and HRMS data; procedures for the dopamine D_2_ functional assays and cytotoxicity experiments in human SH-SY5Y neuronal cells; conformational analysis and Cartesian coordinate tables for compounds **1** and **9** (PDF)

## Notes

This work is dedicated to the memory of Professor Olga Caamaño, in recognition of her contributions and lasting legacy to the medicinal chemistry community.
