# Supplementary material for: Proline Homologation in Melanostatin Neuropeptide: Discovery of Potent Modulators of the Dopamine D2 Receptors
Source: ACS Med Chem Lett. 2025 Jul 24;16(8):1437–44. doi: 10.1021/acsmedchemlett.5c00287 (PMC12358994; doi:10.1021/acsmedchemlett.5c00287)
Supplement: Supplementary file 1 [file ml5c00287_si_001.pdf]

Electronic Supporting Information for:

Proline Homologation in Melanostatin Neuropeptide: Discovery of Potent Modulators of the Dopamine D_2 Receptors

Ivo E. Sampaio-Dias,[a,*] Hugo F. Costa-Almeida,[a,b] Xavier C. Correia,[a] Sara C. Silva-Reis,[a,b] Vera M. Costa,[b,c] Jose Brea,[d] María I. Loza,[d] José E. Rodríguez-Borges,[a] and Xerardo García-Mera[e]

[a]LAQV/REQUIMTE, Department of Chemistry and Biochemistry, Faculty of Sciences, University of Porto, 4169-007 Porto, Portugal.

[b]UCIBIO – Applied Molecular Biosciences Unit, Laboratory of Toxicology, Department of Biological Sciences, Faculty of Pharmacy, University of Porto, 4050-313 Porto, Portugal.

[c]Associate Laboratory i4HB – Institute for Health and Bioeconomy, Laboratory of Toxicology, Department of Biological Sciences, Faculty of Pharmacy, University of Porto, 4050-313 Porto, Portugal.

[d]Innopharma Screening Platform. Biofarma Research Group. Centre of Research in Molecular Medicine and Chronic Diseases (CIMUS), University of Santiago de Compostela, E-15782 Santiago de Compostela, Spain.

[e]Department of Organic Chemistry, Faculty of Pharmacy, University of Santiago de Compostela, E-15782 Santiago de Compostela, Spain.

***Corresponding author e-mail**:
ivdias@fc.up.pt (Ivo E. Sampaio-Dias)

Table of contents

1. Experimental Section: Organic Chemistry S2
   1.1. General Data S2
   1.2. Apparatus S2
   1.3. Synthetic Procedures and Structural Characterization of Compounds **1, 4, 5, 7-9** S3
2. Safety Statement S5
3. Dopamine D_2 Functional Assays S5
4. Cytotoxicity in Human Neuronal SH-SY5Y Cells S6
   4.1. MTT Reduction Assay S7
   4.2. NR Uptake Assay S7
   4.3. Statistical Analysis S7
5. NMR Spectra for Compounds **1, 4, 5, 7-9** S8
6. HRMS Spectra for Compounds **1, 7-9** S18
7. Concentration-Response Curves of Dopamine in the Presence of MIF-1 S20
8. Conformational Analysis and Cartesian Coordinate Tables of Compounds **1** and **9** S20
9. References S25

1. Experimental Section: Organic Chemistry

1.1. General Data. All chemicals described in this work were of reagent grade and used without further purifications: H-Gly-OMe · HCl and TBTU were purchased from Bachem (Bubendorf, Switzerland); Boc-L-Pip-OH, Boc-L-Leu-OSu, and 7 M NH_3 in methanol were obtained from Fluorochem (Derbyshire, UK); TFA was acquired from ABCR GmbH (Karlsruhe, Germany); and Et_3N was purchased from Sigma-Aldrich (Algés, Portugal). High-purity grade silica gel used for flash chromatography (Merk 60A, 230-240 mesh) and pre-coated aluminum silica gel plates used for analytical TLC (Merck 60 F_{254}, 0.25 mm) were obtained from Merck (Darmstadt, Germany). TLC plates were analyzed under UV radiation and/or by using a solution of phosphomolybdic acid in ethanol, requiring gentle heating for visualization. Peptide coupling reactions were conducted under an argon atmosphere.

1.2. Apparatus. Mass spectra were recorded on a Bruker MicrOTOF spectrometer from the Technological Scientific Support Center of the University of Santiago de Compostela (CACTUS). NMR spectra were recorded at Centro de Materiais da Universidade do Porto (CEMUP) with a Bruker Avance III 400 at 400.15 MHz for ^{1}H and 100.62 MHz for ^{13}C. ^{1}H and ^{13}C{^{1}H} chemical shifts are reported in ppm and referenced to the residual protic solvent signals (DMSO-d_6: δ_H = 2.50; CD_3OD: δ_H = 3.31; $CDCl_3$: δ_H = 7.26)[1] in the ^{1}H NMR spectra and to the deuterated solvent signals (DMSO-d_6: δ_H = 39.52; CD_3OD: δ_C = 49.00; $CDCl_3$: δ_C = 77.16)[1] in the ^{13}C{^{1}H} NMR spectra. The assignment of protons and carbons was performed using the three-letter system to specify the α-amino acid residues (Pip: L-pipecolic acid; Leu: L-leucine; Gly: glycine) in subscript, with the indication of the proton (or group of protons) and/or the carbons within a given amino acid residue by starting the numeration at the carbonyl carbon. Determination of the optical rotations was performed on a JASCO P-2000 polarimeter (thermostated), and the data was reported as follows: $\alpha_{\mathrm{D}}^{\theta}$ expressed in (°) (dm^{-1}) (g^{-1}), in which θ is the temperature in Celsius and c (g / 100 mL, solvent). Melting points were determined using a STUART Scientific, model SMP1, and are not corrected. Solvents were evaporated using a Büchi Rotavapor R-210.

1.3. Synthetic Procedures and Structural Characterization of Compounds 1, 4, 5, and 7-9.

1.3.1. Methyl *N*-(*tert*-butyloxycarbonyl)-L-leucylglycinate (4). To a solution of Boc-L-Leu-OSu (2.6400 g, 8.0398 mmol) in CH_2Cl_2 (100 mL) was added Et_3N (3.36 mL, 24.1 mmol), followed by the addition of H-Gly-OMe • HCl (1.1103 g, 8.8435 mmol). The solution was left under magnetic stirring until complete consumption of the starting material (approx. 4 h, TLC). The solvent was then removed, and the residue obtained was dissolved in EtOAc (50 mL), transferred into a separatory funnel, and washed with a saturated aqueous solution of $NaHCO_3$ (*aq*) (2 x 50 mL). The organic phase was then dried over anhydrous Na_2SO_4, filtered, and the filtrate was concentrated using a rotovap. The resulting oil was precipitated by the addition of cold Hex (50 mL) and collected by filtration, affording 2.3556 g (97%) of a white solid. **^{1}H NMR** ($CDCl_3$, 400 MHz) δ ppm: 6.92 (br s, 1H, CONH), 5.09 (d, J = 7.9 Hz, 1H, OCONH), 4.18 (br s, 1H, H_{Leu}-2), 4.08 – 3.92 (m, 2H, H_{Gly}-2), 3.71 (s, 3H, CO_2CH_3), 1.73 – 1.59 (m, 2H, H_{Leu}-3), 1.52 – 1.44 (m, 10H, H_{Leu}-4 + Boc), [0.92 (d, J = 6.2 Hz), 0.90 (d, J = 6.1 Hz), 6H, H_{Leu}-5]. **^{13}C{^{1}H} NMR and DEPT-135** ($CDCl_3$, 101 MHz) δ ppm: [173.2 (C), 170.3 (C), CONH + CO_2CH_3], 155.9 (C, Boc), 80.1 (C, Boc), [53.0, 52.4 (CH + CH_3), CO_2CH_3 + C_{Leu}-2], [41.4 (CH_2), 41.2 (CH_2), C_{Gly}-2 + C_{Leu}-3], 28.4 (3 x CH_3, Boc), 24.8 (CH, C_{Leu}-4), [23.0 (CH_3), 22.0 (CH_3), C_{Leu}-5].

1.3.2. Methyl L-leucylglycinate trifluoroacetate salt (5). To a solution of **4** (2.3128 g, 7.6489 mmol) in anhydrous CH_2Cl_2 (20 mL) was added TFA (17.70 mL, 229.7 mmol), and the solution was left under magnetic stirring until complete consumption of the starting material (4 h, TLC). Then, the volatiles were removed using a rotovap, affording 2.4192 g (100%) of a yellow oil. **^{1}H NMR** (DMSO-d_6, 400 MHz) δ ppm: 8.98 (t, J = 5.8 Hz, 1H, CONH), 8.19 (br s, 3H, $^+NH_3$), 4.00 (dd, J = 17.4, 6.0 Hz, 1H, H_{Gly}-2a), 3.90 (dd, J = 17.4, 5.7 Hz, 1H, H_{Gly}-2b), 3.85 – 3.77 (m, 1H, H_{Leu}-2), 3.64 (s, 3H, CO_2CH_3), [1.77 – 1.64 (m, 1H), 1.64 – 1.46 (m, 2H), H_{Leu}-3 + H_{Leu}-4], [0.91 (d, J = 6.2 Hz), 0.89 (d, J = 6.3 Hz), 6H, H_{Leu}-5]. **^{13}C{^{1}H}-NMR and DEPT-135** (DMSO-d_6, 101 MHz) δ ppm: [169.6 (C), 169.4 (C), CONH + CO_2CH_3], [51.6, 50.5 (CH + CH_3), C_{Leu}-2 + CO_2CH_3], 40.4 (CH_2, C_{Gly}-2), 40.1 (CH_2, C_{Leu}-3), 23.2 (CH, C_{Leu}-4), [22.3 (CH_3), 21.7 (CH_3), C_{Leu}-5].

1.3.3. Methyl *N*-(*tert*-butyloxycarbonyl)-L-pipecolyl-L-leucylglycinate (7). To a solution of **6** (0.5000 g, 2.181 mmol) in CH_2Cl_2 (20 mL) was added Et_3N (0.91 mL, 6.5 mmol), followed by the addition of TBTU (0.7699 g, 2.398 mmol). The suspension was left under magnetic stirring until a clear solution was achieved (20 min). Then, **5** (0.8274 g, 2.616 mmol) was added and the solution was further stirred until completion of the reaction (approx. 3 h, TLC). The solvent was then removed, and the residue obtained was dissolved in EtOAc (20 mL), transferred into a separatory funnel, and washed with a saturated aqueous solution of $NaHCO_3$ (*aq*) (2 x 20 mL). The organic phase was then dried over anhydrous Na_2SO_4, filtered, and the filtrate was concentrated using a rotovap. The crude oil obtained was then purified by chromatography using EtOAc as the eluent,

affording 0.6935 g (77%) of **7** as a white solid. mp = 102-104 ºC. $[\alpha]_D^{25}$ = –78.77 ± 0.11 (*c*1.025, $CHCl_3$). **^{1}H NMR** ($CDCl_3$, 400 MHz) *δ* ppm: 6.83 (br s, 1H, $CONH_{Gly}$), 6.51 (br s, 1H, $CONH_{Leu}$), 4.71 (br s, 1H, H_{Pip}-2), 4.54 – 4.46 (m, 1H, H_{Leu}-2), 4.06 – 3.92 (m, 3H, H_{Gly}-2 + H_{Pip}-6a), 3.72 (s, 3H, CO_2CH_3), 2.77 (t, *J* = 11.2 Hz, 1H, H_{Pip}-6b), 2.16 – 2.12 (m, 1H, H_{Pip}-3a), 1.75 – 1.39 [1.46 (s), 17H, Boc + H_{Pip}-3b + H_{Pip}-4 + H_{Pip}-5 + H_{Leu}-3 + H_{Leu}-4], [0.93 (d, *J* = 6.4 Hz), 0.90 (d, *J* = 6.4 Hz), 6H, H_{Leu}-5]. **^{13}C{^{1}H} NMR and DEPT-135** ($CDCl_3$, 101 MHz) *δ* ppm: [172.2 (C), 171.9 (C), 170.1 (C), 2 x CONH + CO_2CH_3), 156.1 (C, Boc), 81.0 (C, Boc), 54.7 (CH, C_{Pip}-2), 52.4 (CH_3, CO_2CH_3), 51.5 (CH, C_{Leu}-2), 42.3 (CH_2, C_{Pip}-6), 41.2 (CH_2, C_{Gly}-2), 40.8 (CH_2, C_{Leu}-3), 28.4 (3 x CH_3, Boc), 25.6 (CH_2, C_{Pip}-5), 24.9 (CH_2, C_{Pip}-3), 24.9 (CH, C_{Leu}-4), [23.1 (CH_3), 21.9 (CH_3), C_{Leu}-5], 20.6 (CH_2, C_{Pip}-4). HRMS (ESI-TOF) *m*/*z*: $[M + H]^+$ Calcd for $C_{20}H_{36}N_3O_6^+$: 414.2599; Found: 414.2605.

1.3.4. *N*-(*tert*-Butyloxycarbonyl)-L-pipecolyl-L-leucylglycinamide (8). To a round-bottom flask charged with **7** (0.3597 g, 0.8699 mmol) was added a solution of 7 M NH_3 in methanol (25 mL), and the solution was left under magnetic stirring until complete consumption of the starting material (approx. 24 h, TLC). Then, the volatiles were removed using a rotovap, affording 0.3460 g (100%) of **8** as a white solid. mp = 67-70 ºC. $[\alpha]_D^{28}$ = –30.91 ± 0.15 (*c*1.085, CH_3OH). **^{1}H NMR** (CD_3OD, 400 MHz) *δ* ppm: 4.71 (dd, *J* = 6.2, 2.7 Hz, 1H, H_{Pip}-2), 4.41 – 4.35 (m, 1H, H_{Leu}-2), 4.00 – 3.87 [3.97 (d, *J* = 17.1 Hz), 2H, H_{Gly}-2a + H_{Pip}-6a], 3.78 (d, *J* = 17.0 Hz, 1H, H_{Gly}-2b), 3.19 (dt, *J* = 12.6, 3.3 Hz, 1H, H_{Pip}-6b), 2.20 (d, *J* = 13.7 Hz, 1H, H_{Pip}-3a), 1.80 – 1.63 (m, 6H, H_{Pip}-3b + H_{Pip}-4a + H_{Pip}-5a + H_{Leu}-3 + H_{Leu}-4), 1.57 – 1.38 [1.50 (s), 11H, Boc + H_{Pip}-4b + H_{Pip}-5b], [1.02 (d, *J* = 6.4 Hz), 0.99 (d, *J* = 6.4 Hz), 6H, H_{Leu}-5]. **^{13}C{^{1}H} NMR and DEPT-135** (CD_3OD, 101 MHz) *δ* ppm: [175.4 (C), 175.0 (C), 174.2 (C), 2 x CONH + $CONH_2$), 157.7 (C, OCON), 81.5 (C, Boc), 55.9 (CH, C_{Pip}-2), 53.6 (CH, C_{Leu}-2), 43.5 (CH_2, C_{Pip}-6), 43.2 (CH_2, C_{Gly}-2), 41.4 (CH_2, C_{Leu}-3), 28.6 (3 x CH_3, Boc), 28.3 (CH_2, C_{Pip}-3), 25.9 (CH, C_{Leu}-4), 25.7 (CH_2, C_{Pip}-3), [23.4 (CH_3), 22.0 (CH_3), C_{Leu}-5], 21.2 (CH_2, C_{Pip}-4). HRMS (ESI-TOF) *m*/*z*: $[M + H]^+$ Calcd for $C_{19}H_{35}N_4O_5^+$: 399.2602; Found: 399.2604.

1.3.5. Methyl L-pipecolyl-L-leucylglycinate trifluoroacetate (9). To a solution of **7** (0.1001 g, 0.2421 mmol) in CH_2Cl_2 (20 mL) was added TFA (0.56 mL, 7.3 mmol), and the solution was left under magnetic stirring until complete consumption of the starting material (4 h, TLC). Then, the volatiles were removed using a rotovap, and the crude oil obtained was purified by chromatography using CH_2Cl_2/CH_3OH (5:1) as the eluent, affording 0.1004 g (97%) of **9** as a white solid. mp = 144-146 ºC. $[\alpha]_D^{27}$ = +84.79 ± 0.17 (*c*1.010, CH_3OH). **^{1}H NMR** (CD_3OD, 400 MHz) *δ* ppm: 4.52 (t, *J* = 7.6 Hz, H_{Leu}-2), 4.03 (d, *J* = 17.6 Hz, 1H, H_{Gly}-2a), 3.95 – 3.83 [3.91 (d, *J* = 17.7 Hz), 2H, H_{Gly}-2b + H_{Pip}-2], 3.75 (s, 3H, CO_2CH_3), [3.47 – 3.38 (m, 1H), 3.06 (dt, *J* = 12.6, 3.4 Hz, 1H), H_{Pip}-6], 2.35 – 2.23 (m, 1H, H_{Pip}-3a), 1.99 – 1.88 (m, 2H, H_{Pip}-4a + H_{Pip}-5a), 1.82 – 1.62 (m, 6H, H_{Pip}-3b + H_{Pip}-4b + H_{Pip}-5b + H_{Leu}-3 + H_{Leu}-4), [1.01 (d, *J* = 6.6 Hz), 0.98 (d, *J* = 6.5 Hz), 6H, H_{Leu}-5]. **^{13}C{^{1}H} NMR and DEPT-**

135 (CD_3OD, 101 MHz) *δ* ppm: [174.8 (C), 171.5 (C), 170.0 (C), 2 x CONH + CO_2CH_3], 59.0 (CH, C_{Pip}-2), 53.1 (CH, C_{Leu}-2), 52.6 (CH_3, CO_2CH_3), 45.0 (CH_2, C_{Pip}-6), 42.0 (CH_2, C_{Leu}-3), 41.8 (CH_2, C_{Gly}-2), 28.6 (CH_2, C_{Pip}-3), 25.8 (CH, C_{Leu}-4), 23.3 (CH_3, C_{Leu}-5a), [23.0 (CH_2), 22.8 (CH_2), C_{Pip}-3 + C_{Pip}-4], 22.0 (CH_3, C_{Leu}-5b). HRMS (ESI-TOF) *m*/*z*: $[M + H]^+$ Calcd for $C_{15}H_{28}N_3O_4^+$: 314.2074; Found: 314.2078.

1.3.6. L-Pipecolyl-L-leucylglycinamide trifluoroacetate (1). To a solution of **8** (0.2854 g, 0.7162 mmol) in CH_2Cl_2 (20 mL) was added TFA (1.83 mL, 23.8 mmol), and the solution was left under magnetic stirring until complete consumption of the starting material (2 h, TLC). Then, the volatiles were removed using a rotovap, and the crude oil obtained was purified by chromatography using EtOAc followed by EtOAc/CH_3OH (9:1) as the eluent, affording 0.2245 g (76%) of **1** as a white solid. mp = 145-148 ºC. $[\alpha]_D^{27}$ = +5.80 ± 0.19 (*c*1.010, CH_3OH). **^{1}H NMR** (CD_3OD, 400 MHz) *δ* ppm: 4.39 (t, *J* = 7.5 Hz, H_{Leu}-2), 3.90 (d, *J* = 17.0 Hz, 1H, H_{Gly}-2a), 3.86 – 3.80 (m, 1H, H_{Pip}-2), 3.77 (d, *J* = 17.0 Hz, 1H, H_{Gly}-2b), [3.43 – 3.36 (m, 1H), 3.02 (dt, *J* = 12.5, 3.4 Hz, 1H), H_{Pro}-6], 2.33 – 2.22 (m, 1H, H_{Pip}-3a), 1.97 – 1.83 (m, 2H, H_{Pip}-4a + H_{Pip}-5a), 1.77 – 1.59 (m, 6H, H_{Pip}-3b + H_{Pip}-4b + H_{Pip}-5b + H_{Leu}-3 + H_{Leu}-4), [0.98 (d, *J* = 6.4 Hz), 0.95 (d, *J* = 6.4 Hz), 6H, H_{Leu}-5]. **^{13}C{^{1}H} NMR and DEPT-135** (CD_3OD, 101 MHz) *δ* ppm: [174.7 (C), 174.0 (C), 170.4 (C), 2 x CONH + $CONH_2$], 59.0 (CH, C_{Pip}-2), 53.7 (CH, C_{Leu}-2), 45.0 (CH_2, C_{Pip}-6), 43.1 (CH_2, C_{Leu}-3), 41.5 (CH_2, C_{Gly}-2), 28.5 (CH_2, C_{Pip}-3), 25.8 (CH, C_{Leu}-4), 23.3 (CH_3, C_{Leu}-5a), [23.0 (CH_2), 22.8 (CH_2), C_{Pip}-4 + C_{Pip}-5], 22.0 (CH_3, C_{Leu}-5b). HRMS (ESI-TOF) *m*/*z*: $[M + H]^+$ Calcd for $C_{14}H_{27}N_4O_3^+$: 299.2078; Found: 299.2079.

2. Safety Statement.
No unexpected or unusually high safety hazards were encountered.

3. Dopamine D_2 Functional Assays.
Functional assays were conducted in CHO cells expressing human D_2R using a Cisbio cAMP kit for the detection of cAMP changes through HTRF technology, as previously described.[2-4] In brief, 5,000 cells per well were seeded in a 96-well black plate containing stimB buffer (provided with the kit) and 500 µM IBMX (a pan-phosphodiesterase inhibitor) to ensure significant cAMP accumulation. Following this, test compounds and dopamine (DA) were added and incubated for 10 minutes at 37 °C, followed by an additional 5-minute incubation with 10 µM forskolin. After the addition of the kit reagents and a 1-hour incubation at room temperature, the HTRF signal was measured using a Tecan M1000 Pro multilabel reader. The data were converted to cAMP using a standard cAMP curve, and the activity of the test compounds was determined as the percentage increase in the activity of 0.1 µM DA. A DA curve was included as the positive control in all assays.

4. Cytotoxicity in Human Neuronal SH-SY5Y Cells. The SH-SY5Y cell line was sourced from Sigma-Aldrich (Taufkirchen, Germany). DMEM medium [DMEM (1x) + GlutaMAX], DMEM/F-12, and Fetal Bovine Serum (FBS) were purchased from Gibco and Alfagene (Carcavelos, Portugal). Penicillin and Streptomycin were obtained from Biotecnómica (Porto, Portugal). Phosphate-buffered solution (PBS) without calcium and magnesium was acquired from Biochrom (Berlin, Germany). Trypsin/EDTA solution, 0.4% (w/v) trypan blue solution, NR solution, RA, and TPA were also obtained from Sigma-Aldrich (Germany). DMSO and HBSS (Hanks' Balanced Salt Solution) were purchased from Merck (Germany). MTT was sourced from Alfa Aesar (Kandel, Germany). 6-OHDA hydrobromide (CAS: 636-00-0) was acquired from Sigma-Aldrich (Germany). Cell counts were performed on a Neubauer chamber using a Nikon Eclipse TS100 microscope from Nikon (Japan), and plate readings (MTT reduction and NR uptake assays) were conducted on a BioTek Synergy HT plate reader from BioTek (USA). Tissue culture flasks (25 cm²) and tissue culture 48-well plates were purchased from TPP (Switzerland), while 5-, 10-, and 25-mL serological pipettes were obtained from Nerbe-Plus (Germany). Throughout all procedures, SH-SY5Y cells were maintained in a 5% CO_2 incubator from Heraeus (Germany) at 37 ºC. Cells were trypsinized after reaching confluence before experiments or for general culture maintenance.

Human neuroblastoma SH-SY5Y cells were maintained in complete DMEM medium supplemented with 10% (v/v) FBS and 1% (v/v) antibiotics (100 units/mL penicillin and 100 µg/mL streptomycin), at 37 °C in a 5% CO_2 incubator throughout all procedures. For the plate seeding, cells were first washed with PBS (devoid of calcium and magnesium), then trypsinized with trypsin/EDTA solution. After trypsin inactivation, cells were counted using a 0.4% (w/v) trypan blue solution and an optical microscope. The cell suspension was then seeded in multiwell plates at a density of 25,000 cells/cm² in complete DMEM containing 10 µM RA for 3 days. On day 3, cells were exposed to 80 nM TPA in complete DMEM and incubated for another 3 days to develop a dopaminergic phenotype.[2, 3, 5] On day 6, after the differentiation protocol, cells were treated with MIF-1 and the test compounds (**1**,**7-9**) at 100 µM, along with 6-OHDA at 125 µM for 48 h in fresh complete DMEM. Here, 6-OHDA was used as a neurotoxicant to mimic the PD-like phenotype. [2, 3, 5] All test compounds were dissolved in sterile PBS before incubation.

4.1. MTT Reduction Assay. In this assay, the tetrazolium MTT salt is reduced to the corresponding formazan by dehydrogenases, predominantly mitochondrial.[6] The protocol was performed as follows: after the 48 h incubation, the cellular medium was replaced with new pre-warmed complete DMEM with MTT at 0.5 mg/mL final concentration, followed by a 1.5 h incubation period at 37 °C with 5% CO_2. The reaction was then stopped by removing the medium and adding DMSO to solubilize the formazan crystals. This was followed by 15 min of agitation protected from light. The absorbance was measured at 570 and 690 nm (reference) in a multiwell plate reader [BioTek Synergy HT (Winooski, VT, USA)]. The values are expressed as a percentage of control cells that were set to 100%, after subtracting the reference absorbance value of each well. All conditions were performed at least in quadruplicate in each independent experiment.

4.2. NR Uptake Assay. This assay relies on the ability of viable cells to uptake NR, a eurhodin dye that accumulates in lysosomes due to their lower internal pH.[6, 7] The protocol was performed as follows: after the 48 h incubation the cellular medium was changed for new pre-warm complete DMEM containing 33 μg/mL of NR, followed by a 1.5 h incubation period at 37 °C with 5% CO_2. Then, the NR medium was removed, and the cells were washed with 250 μL of HBSS with calcium and magnesium. After removing the HBSS solution, 200 μL of NR desorbent ethanol/acetic acid solution (50% ethanol, 1% acetic acid, 49% H_2O) was added. Plates were left under agitation and protected from light for 15 min. Then, the absorbance was measured at 540 and 690 nm (reference) in a multiwell plate reader [BioTek Synergy HT (Winooski, VT, USA)]. The values are expressed as a percentage of control cells that were set to 100%, after subtracting the reference absorbance value of each well. All conditions were performed in quadruplicate in each independent experiment.

4.3. Statistical Analysis. The results are expressed as mean ± standard deviation. An ordinary one-way ANOVA was performed, followed by Tukey's *post hoc* test when a significant *p*-value was reached ($p < 0.05$). The GraphPad Prism 8.3 software (CA, USA) was used to perform all statistical analyses.

5. NMR Spectra for Compounds 1, 4, 5, 7-9

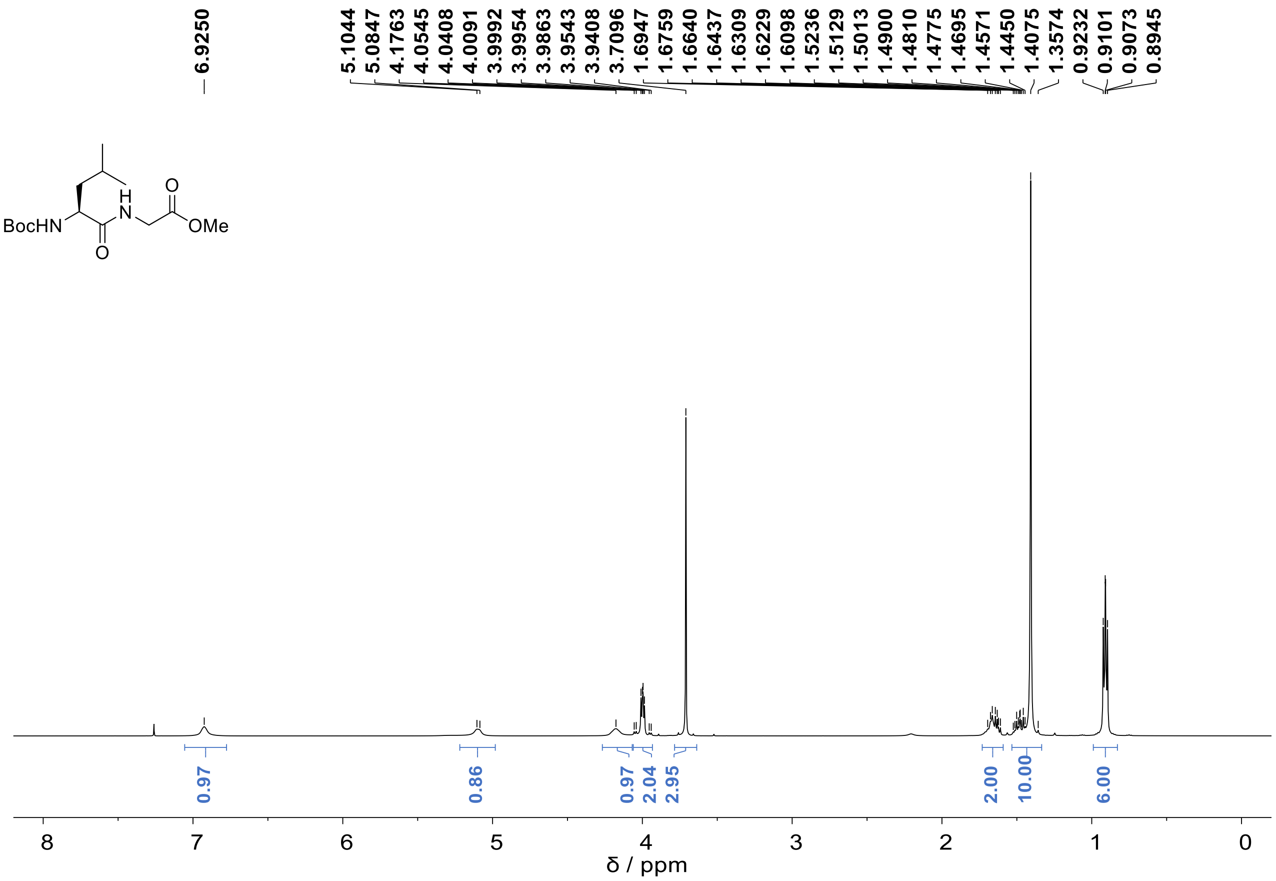

Figure S1. ^{1}H NMR spectrum ($CDCl_3$, 400 MHz) of **4**.

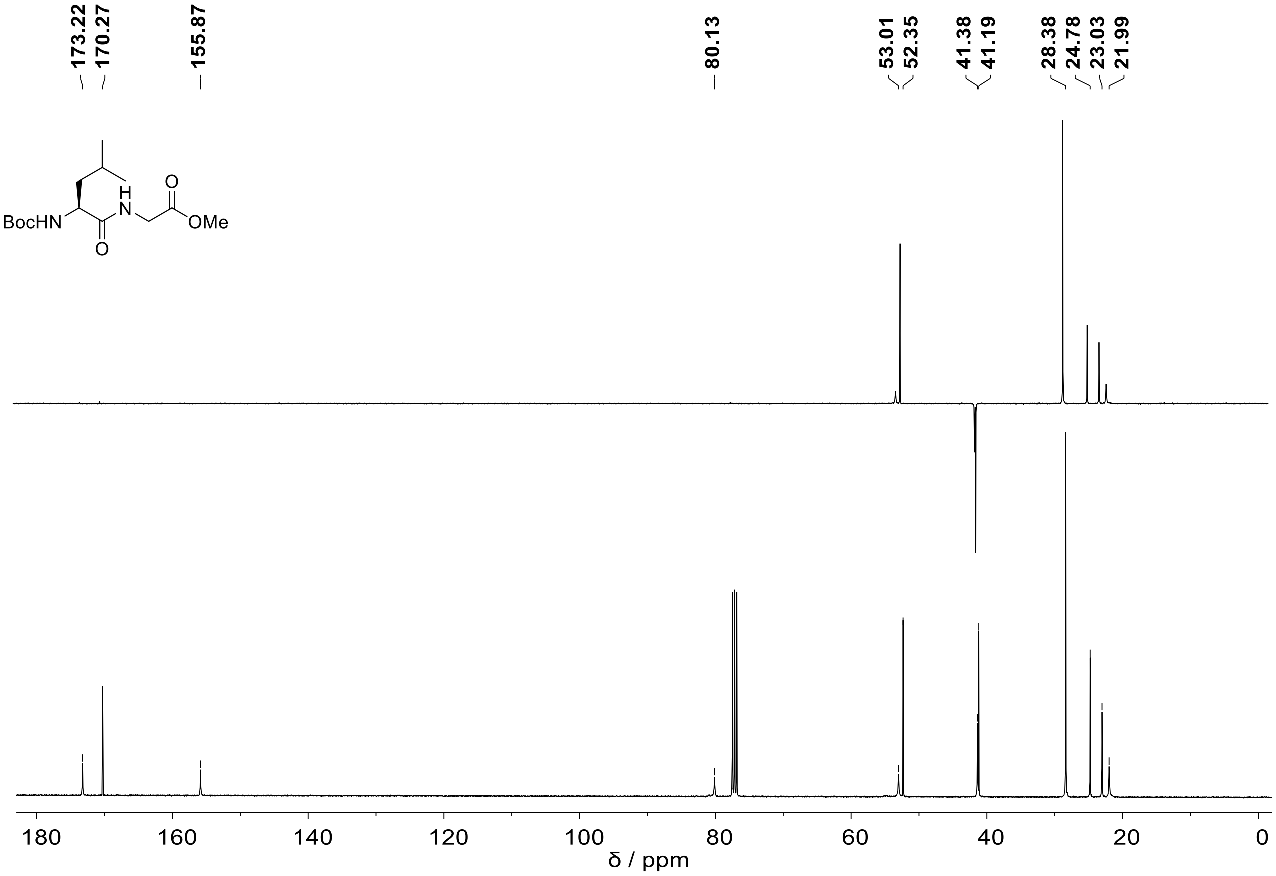

Figure S2. $^{13}C\{^{1}H\}$ and DEPT-135 NMR spectra ($CDCl_3$, 100 MHz) of **4**.

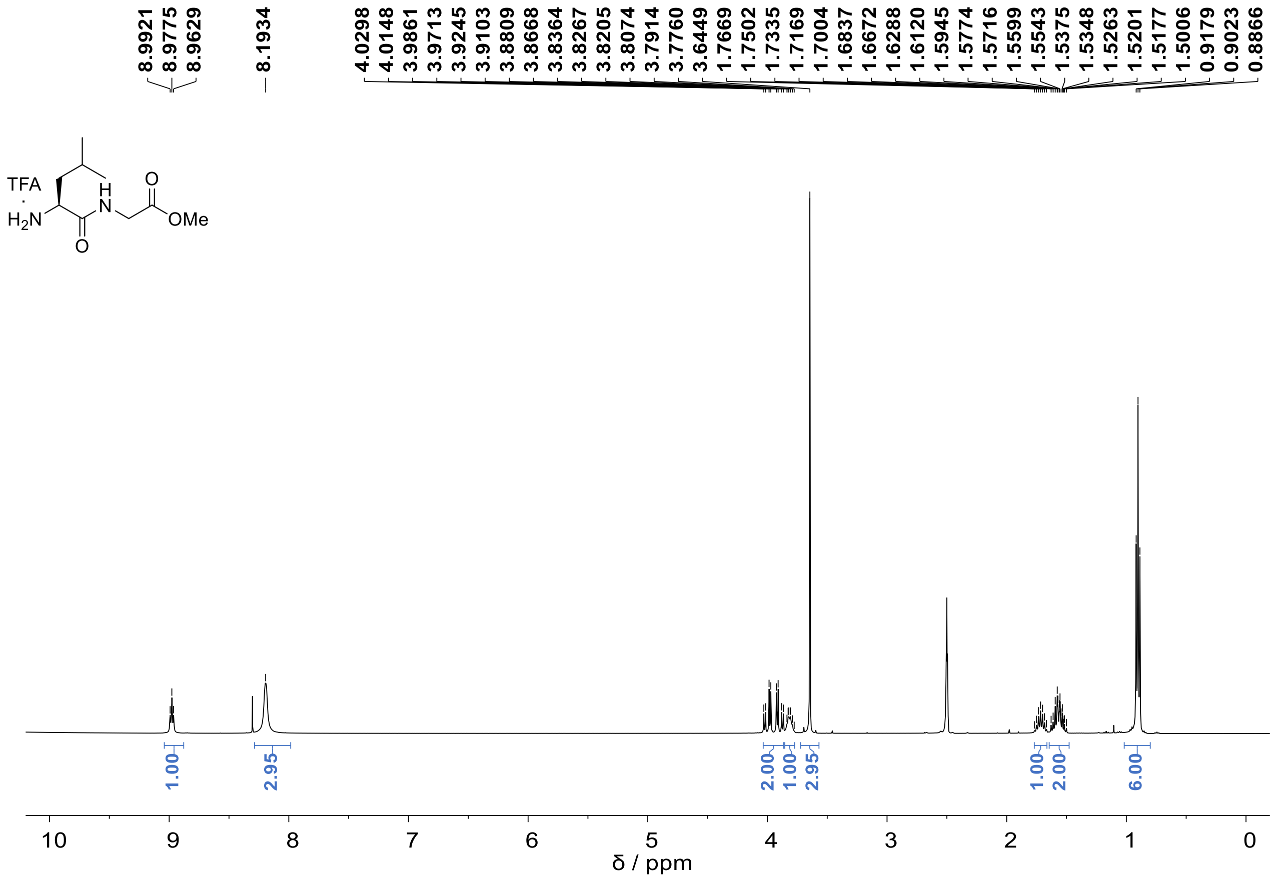

Figure S3. ^{1}H NMR spectrum (DMSO-d_6, 400 MHz) of **5**.

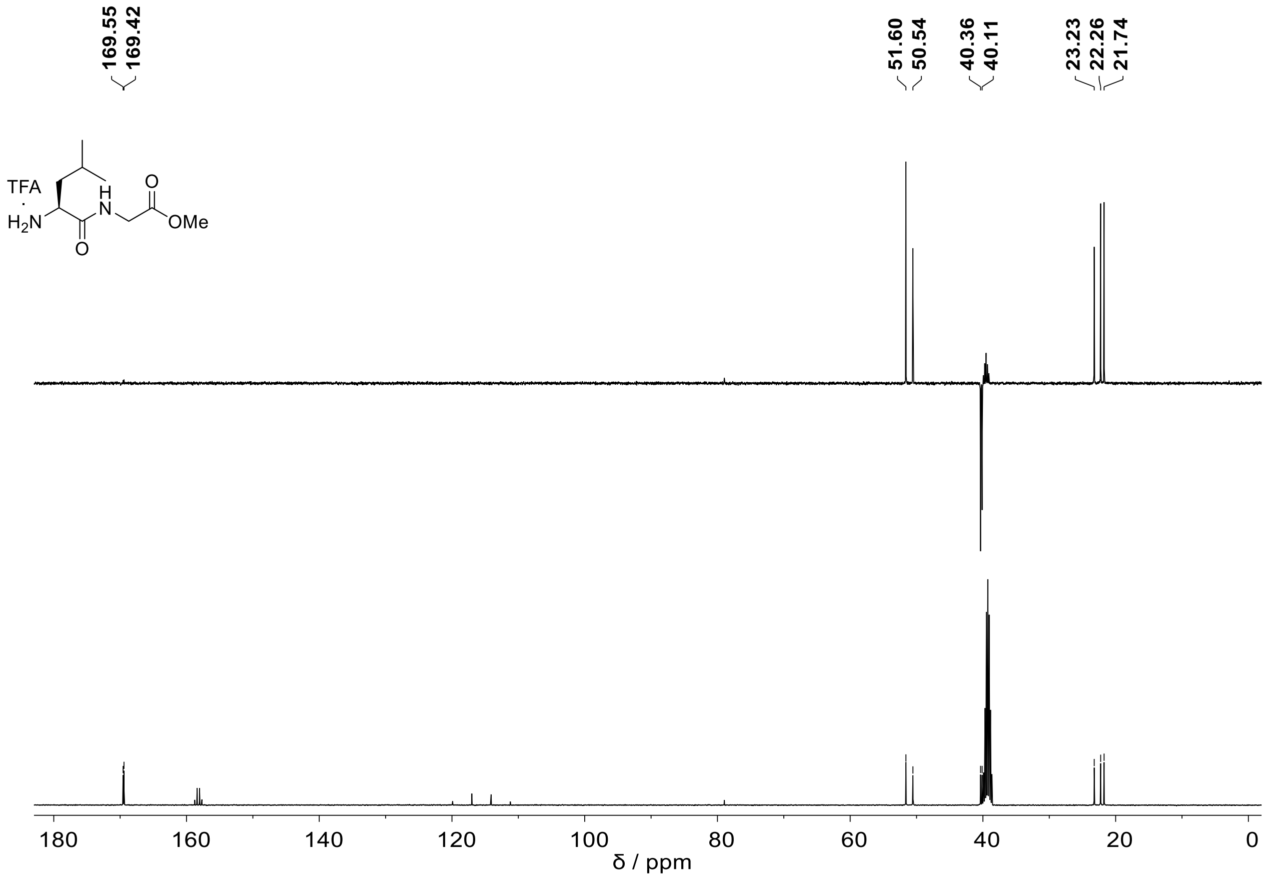

Figure S4. ^{13}C{^{1}H} and DEPT-135 NMR spectra (DMSO-d_6, 100 MHz) of **5**.

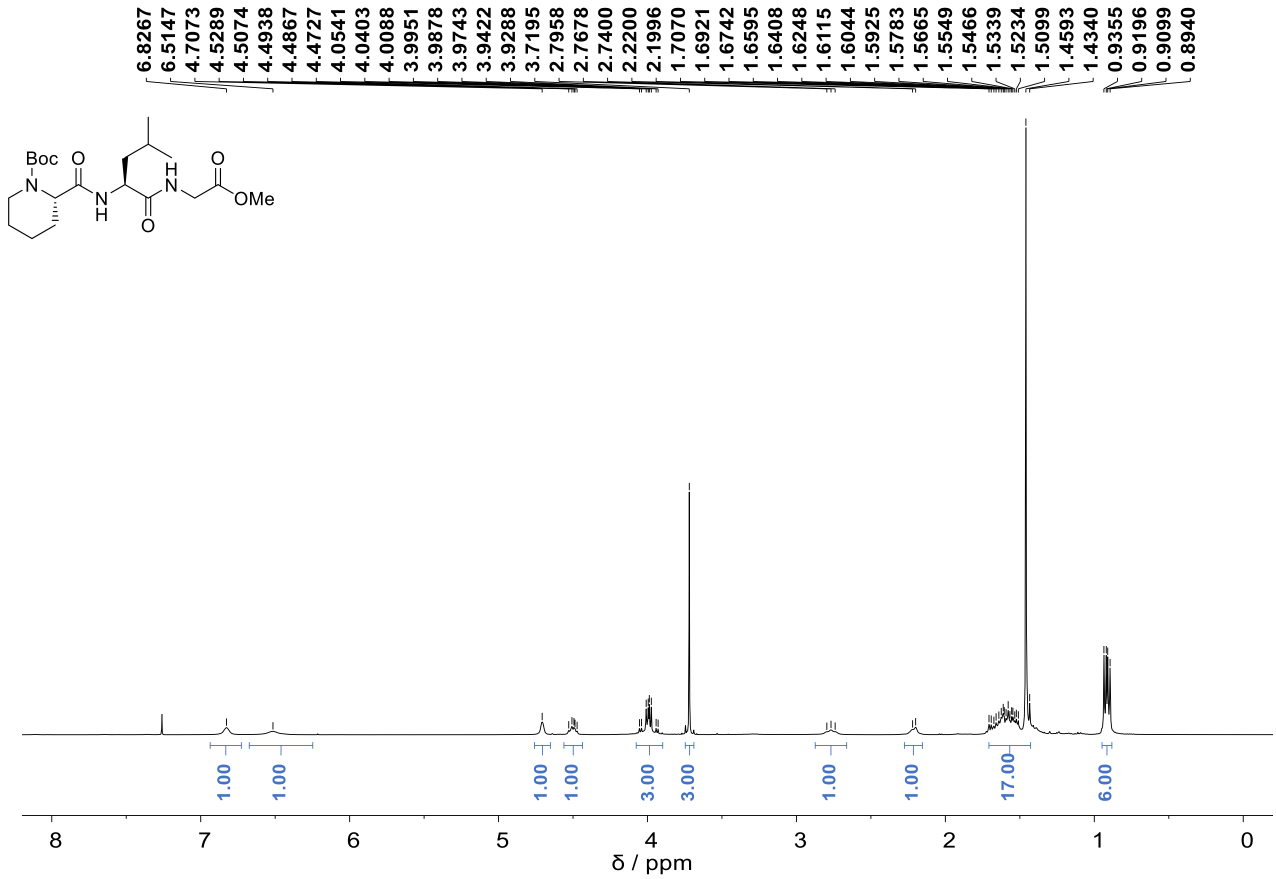

Figure S5. ^{1}H NMR spectrum ($CDCl_3$, 400 MHz) of **7**.

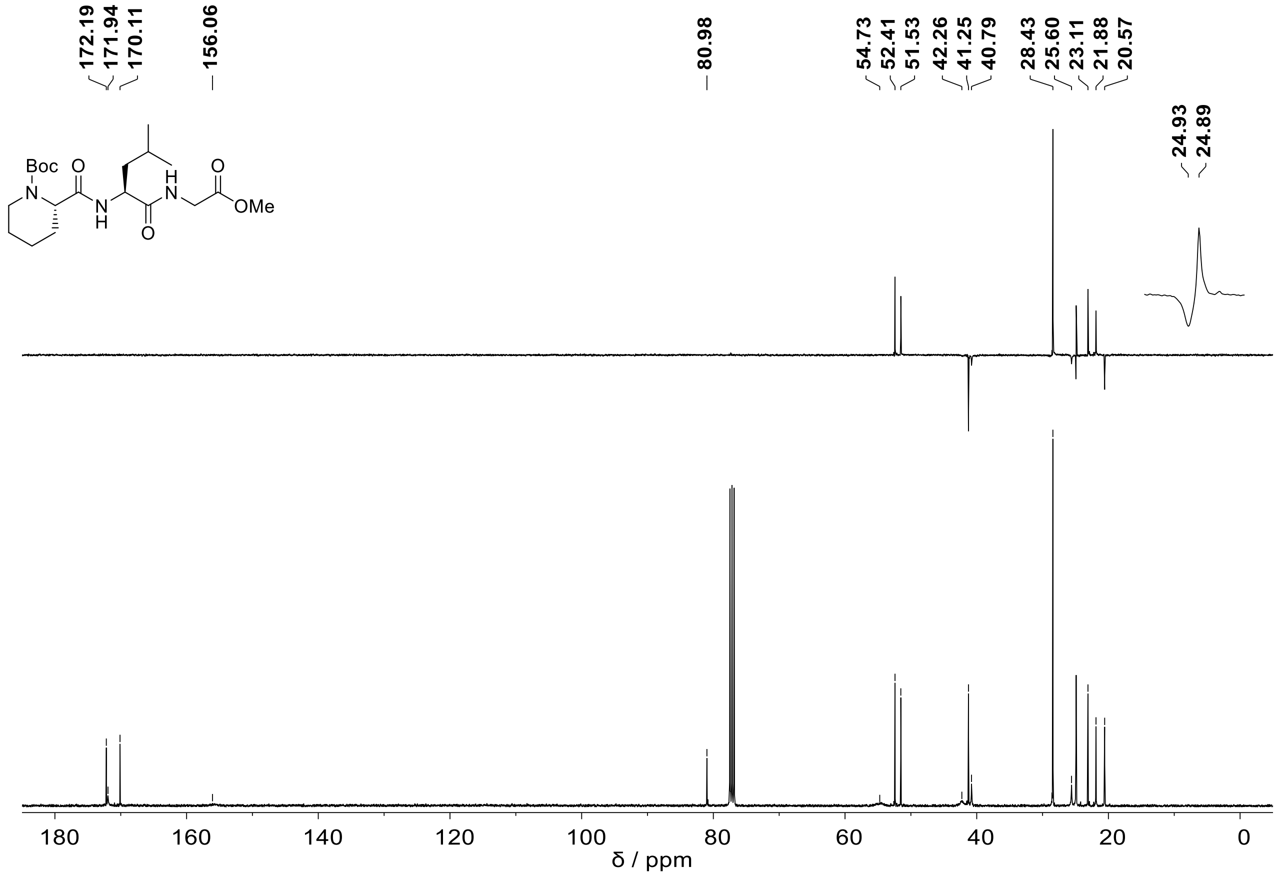

Figure S6. $^{13}C\{^{1}H\}$ and DEPT-135 NMR spectra ($CDCl_3$, 100 MHz) of **7**.

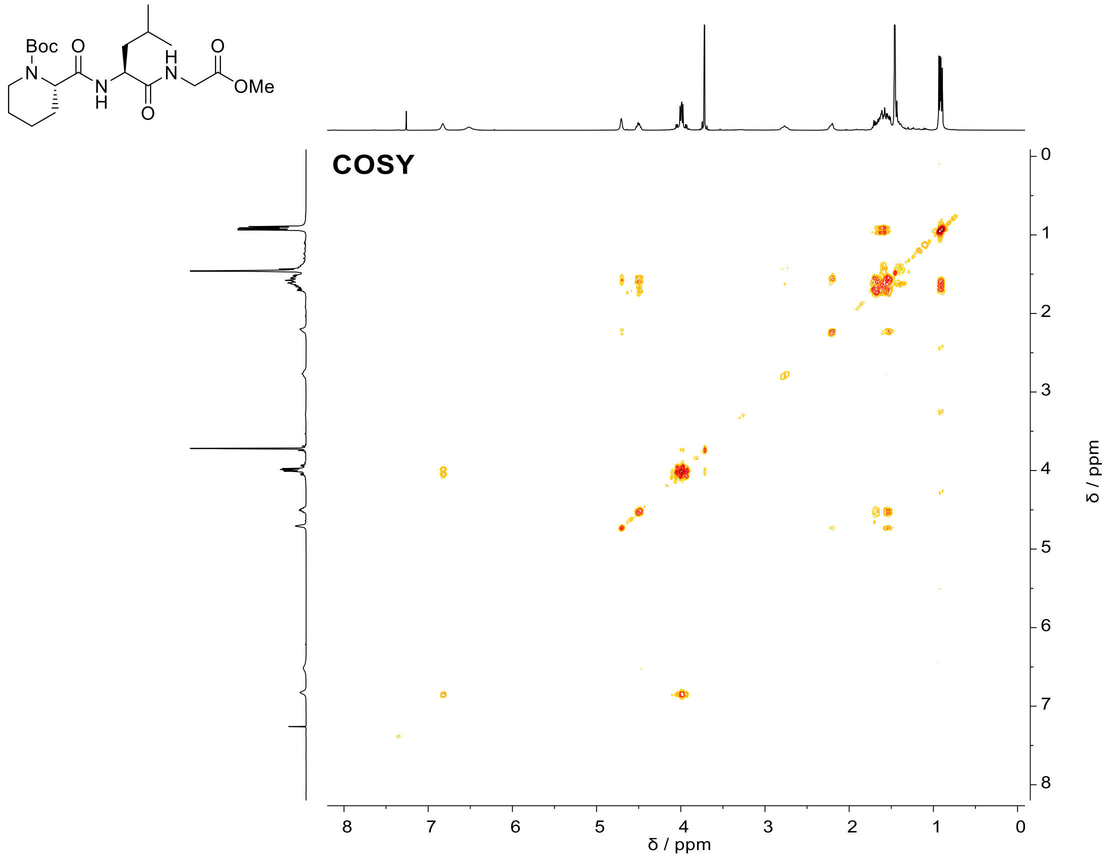

Figure S7. ^{1}H-^{1}H NMR (COSY) spectrum ($CDCl_3$, 400 MHz) of **7**.

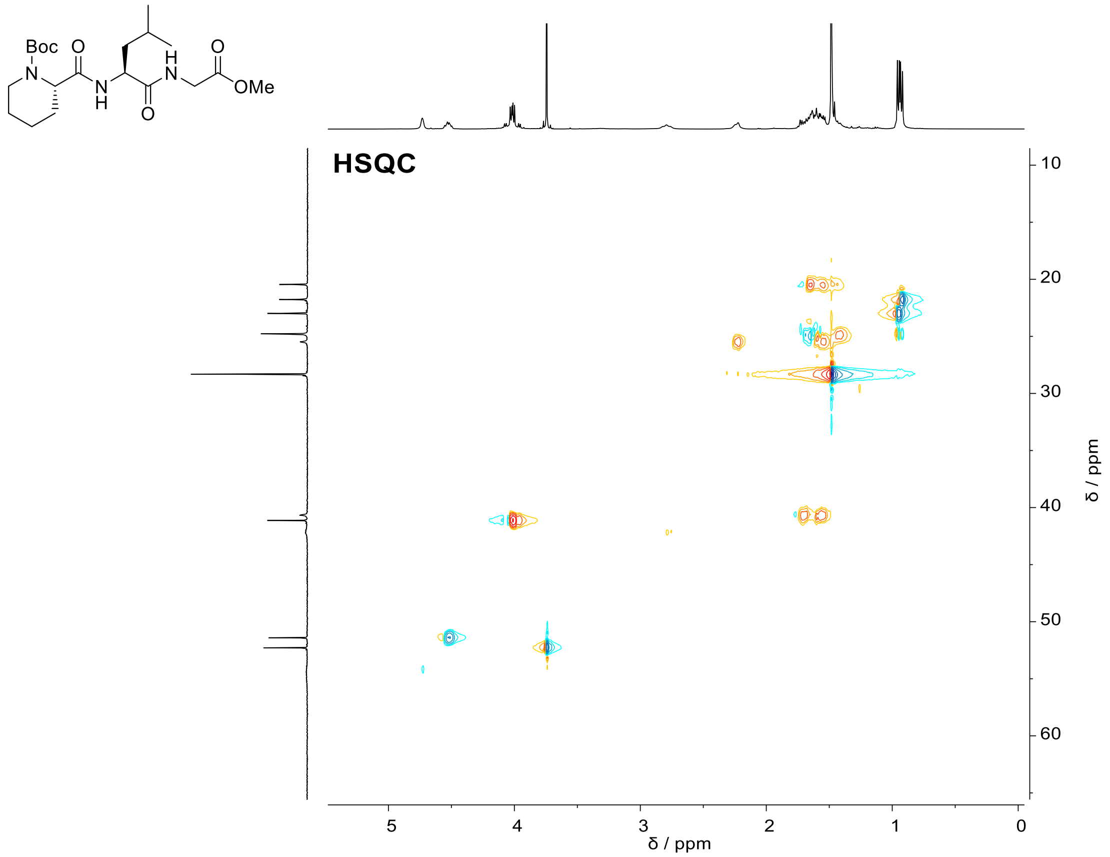

Figure S8. ^{1}H-^{13}C NMR (HSQC) spectrum ($CDCl_3$, 400 MHz) of **7**.

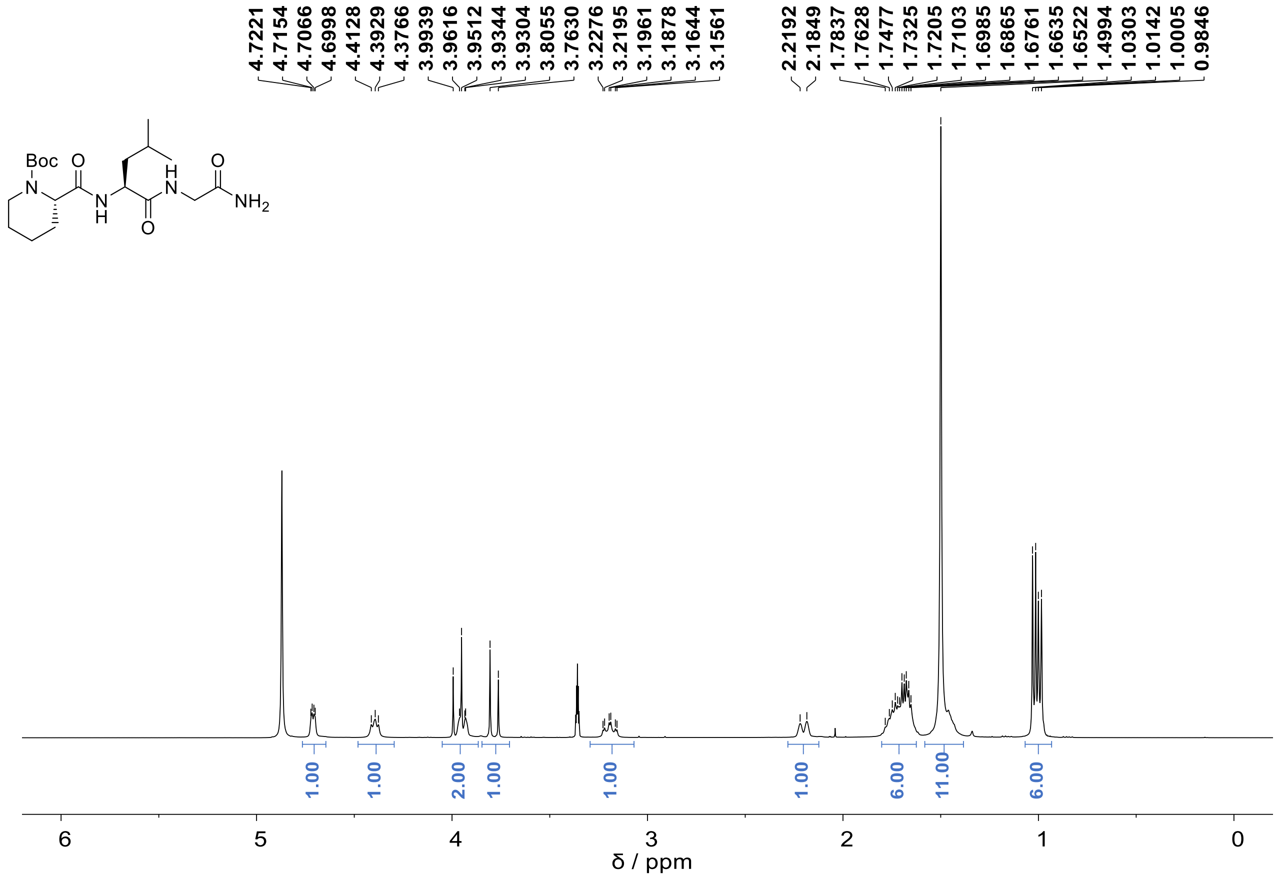

Figure S9. ^{1}H NMR spectrum (CD_3OD, 400 MHz) of **8**.

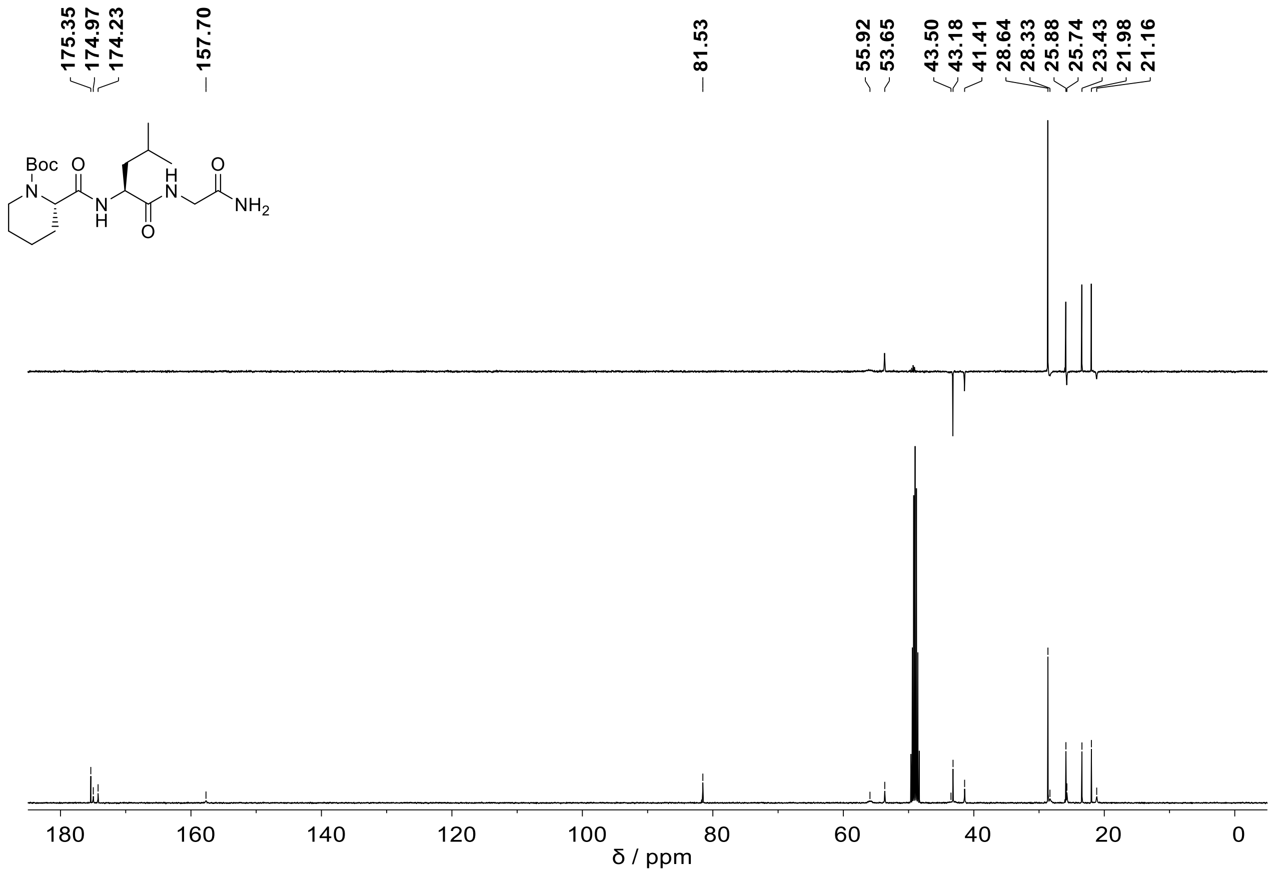

Figure S10. $^{13}C\{^1H\}$ and DEPT-135 NMR spectra (CD_3OD, 100 MHz) of **8**.

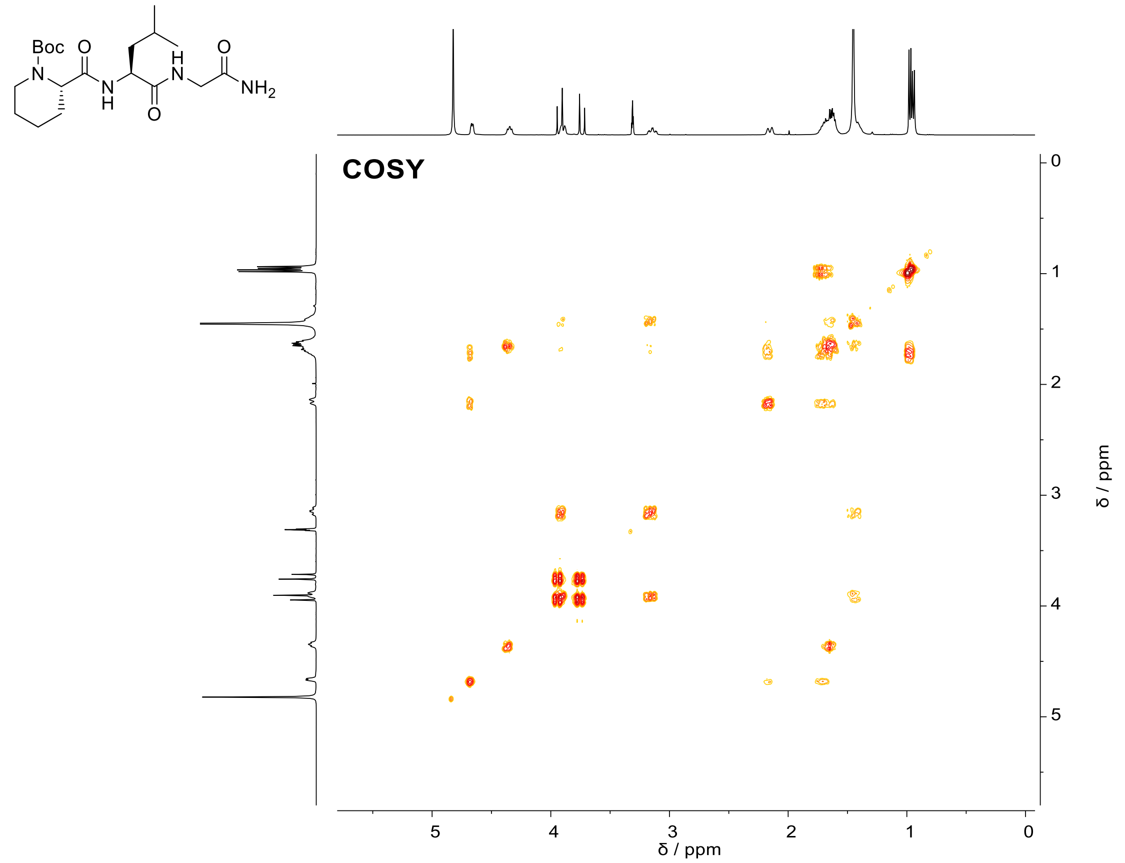

Figure S11. ^{1}H-^{1}H NMR (COSY) spectrum (CD_3OD, 400 MHz) of **8**.

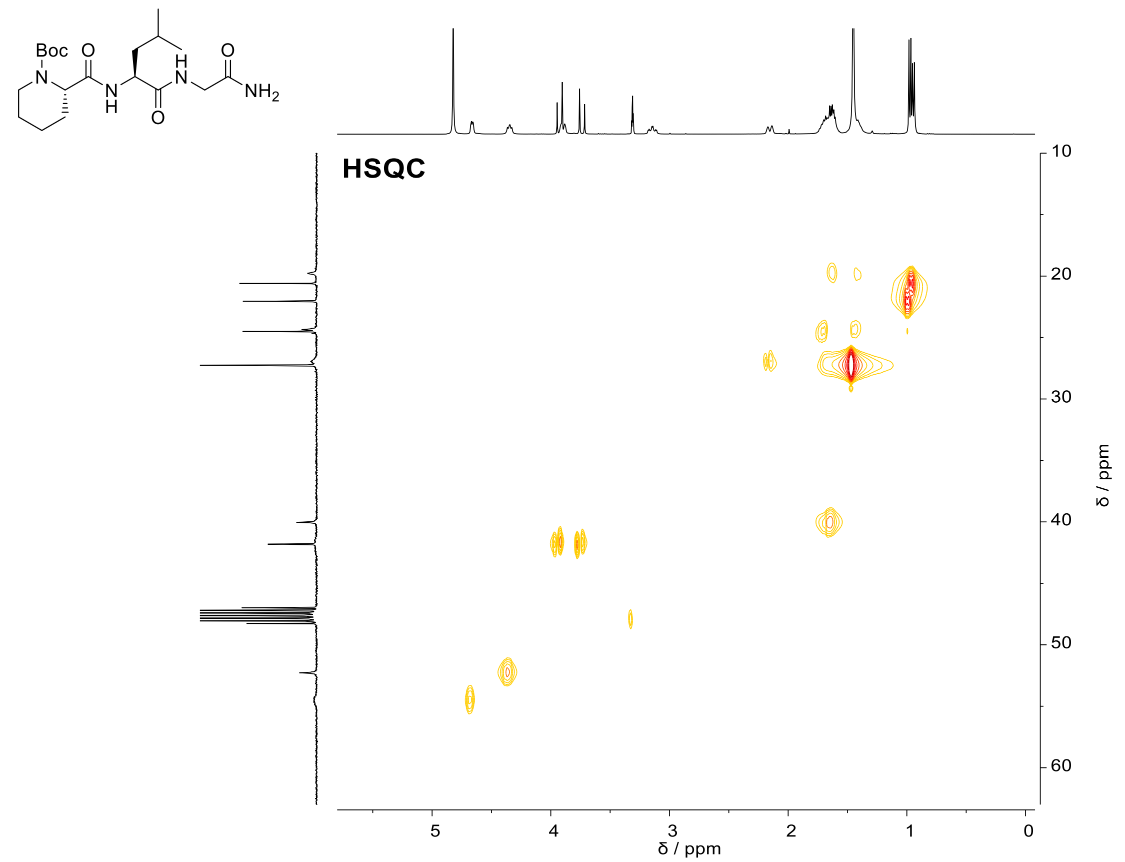

Figure S12. ^{1}H-^{13}C NMR (HSQC) spectrum (CD_3OD, 400 MHz) of **8**.

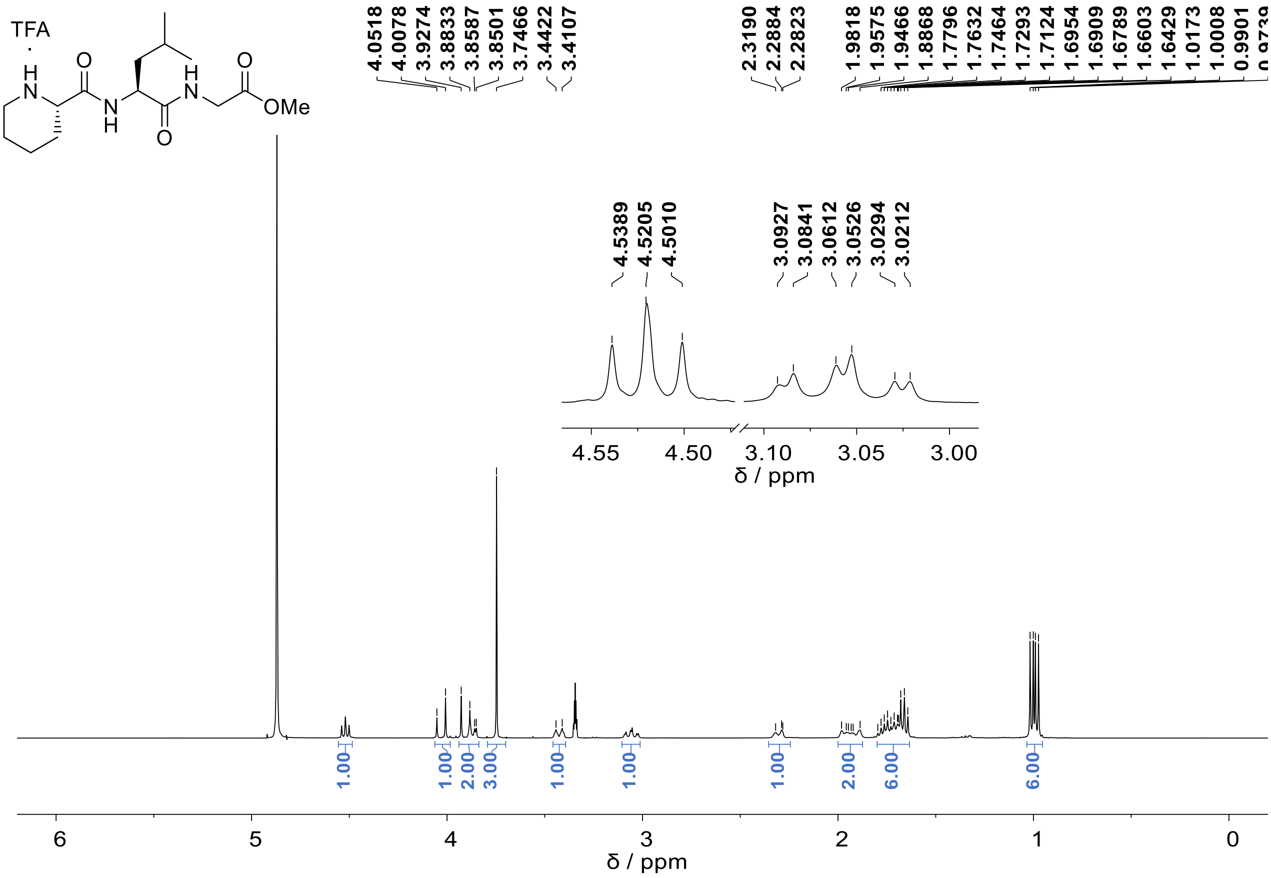

Figure S13. ^{1}H NMR spectrum (CD_3OD, 400 MHz) of **9**.

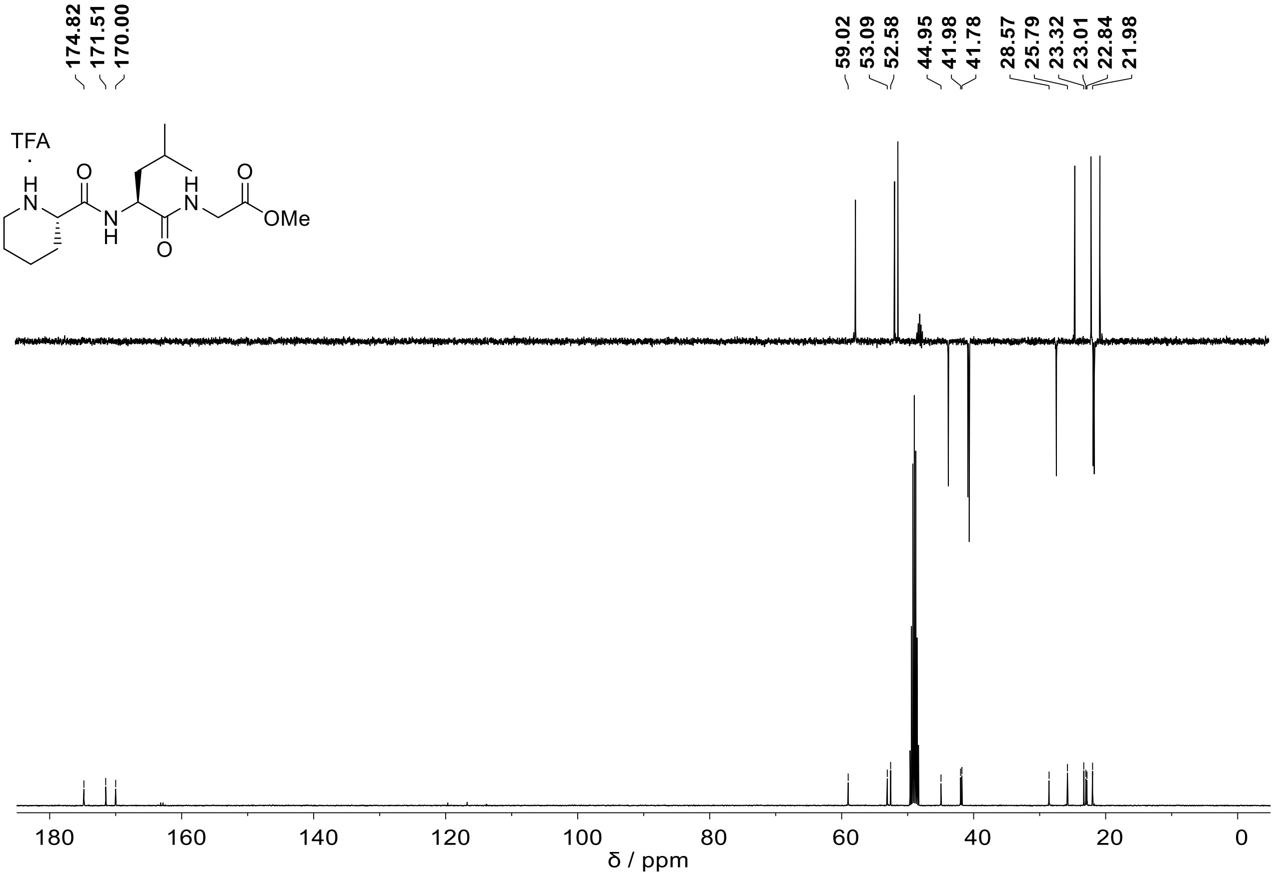

Figure S14. $^{13}C\{^{1}H\}$ and DEPT-135 NMR spectra (CD_3OD, 100 MHz) of **9**.

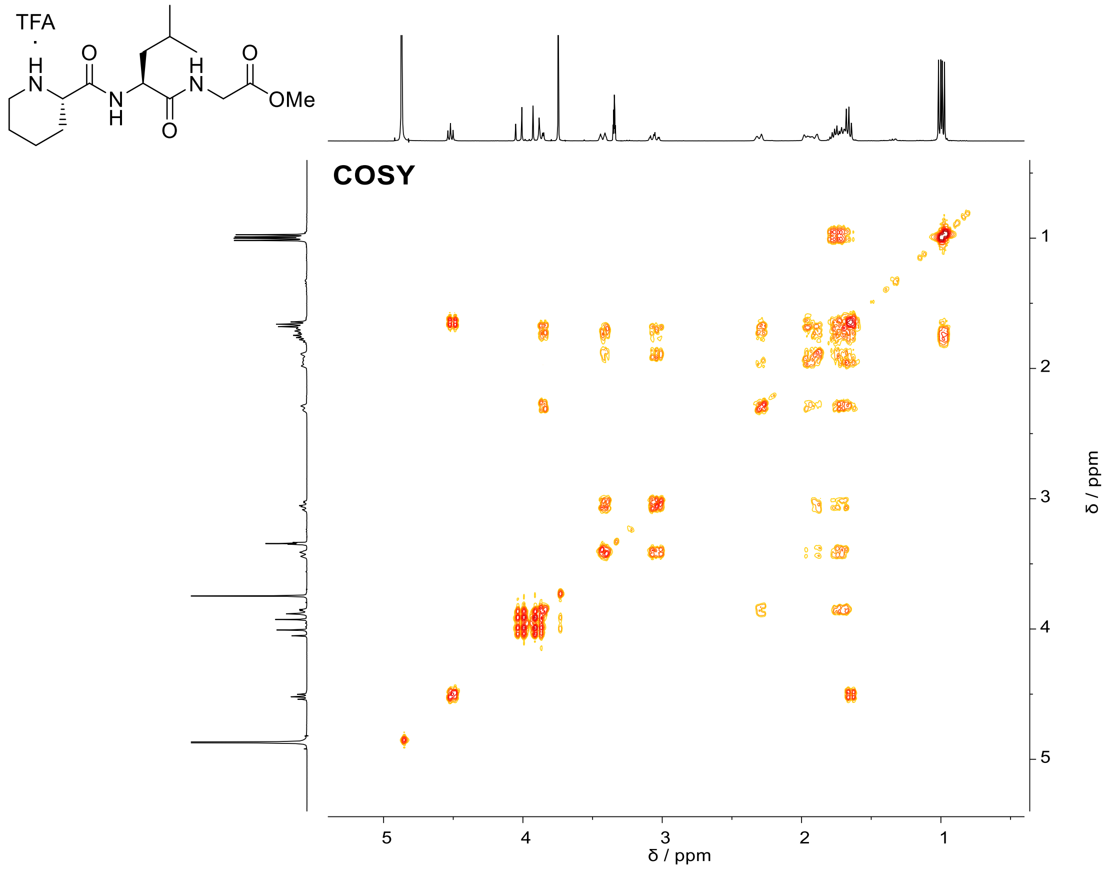

Figure S15. ¹H-¹H NMR (COSY) spectrum (CD_3OD, 400 MHz) of **9**.

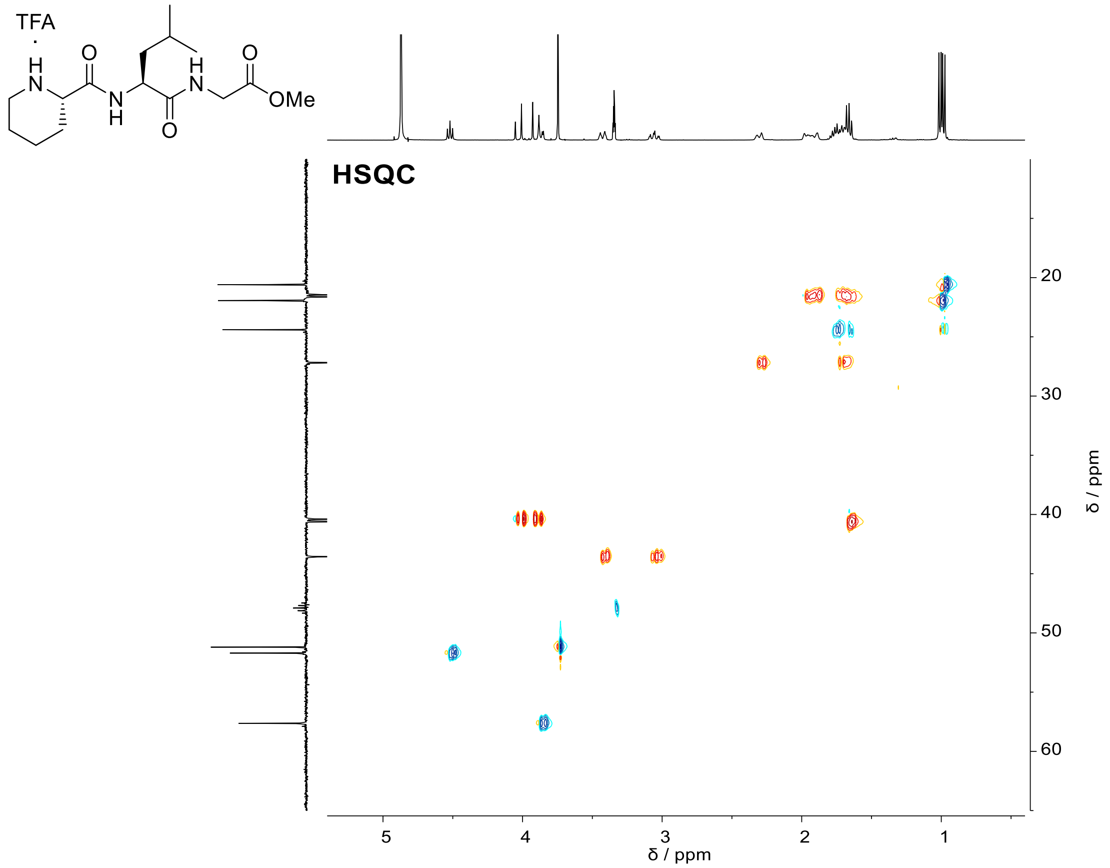

Figure S16. ¹H-¹³C NMR (HSQC) spectrum (CD_3OD, 400 MHz) of **9**.

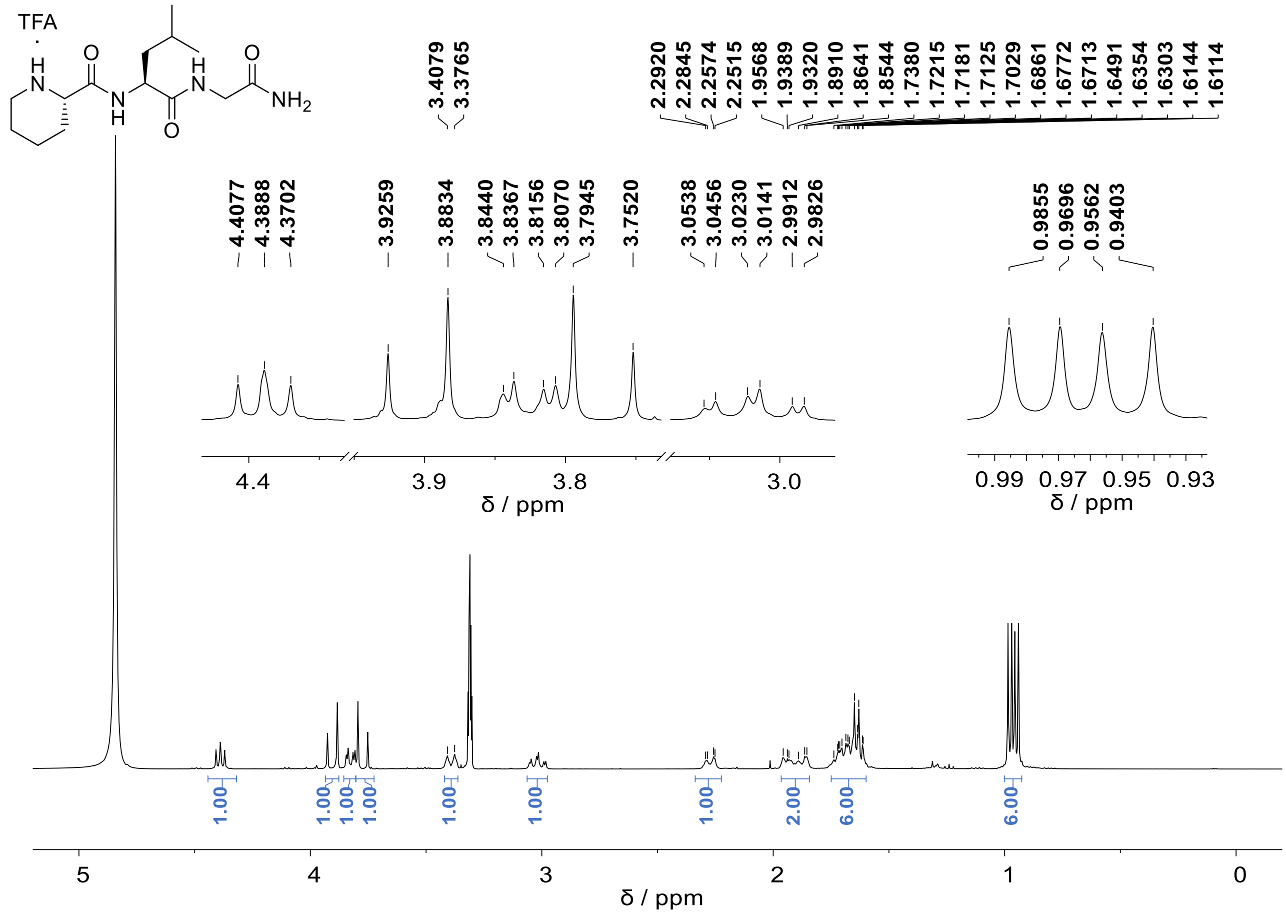

Figure S17. ^{1}H NMR spectrum (CD_3OD, 400 MHz) of **1**.

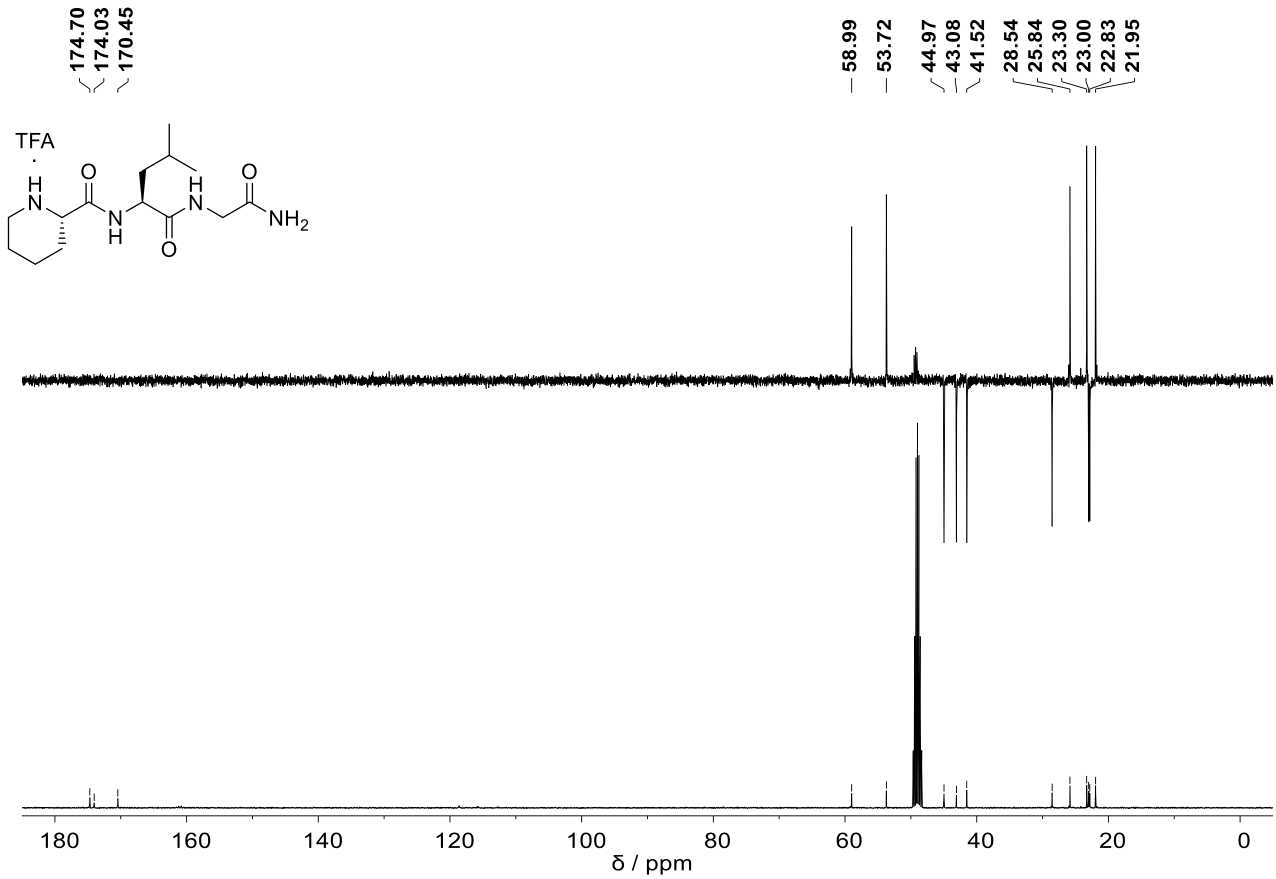

Figure S18. $^{13}C\{^{1}H\}$ and DEPT-135 NMR spectra (CD_3OD, 100 MHz) of **1**.

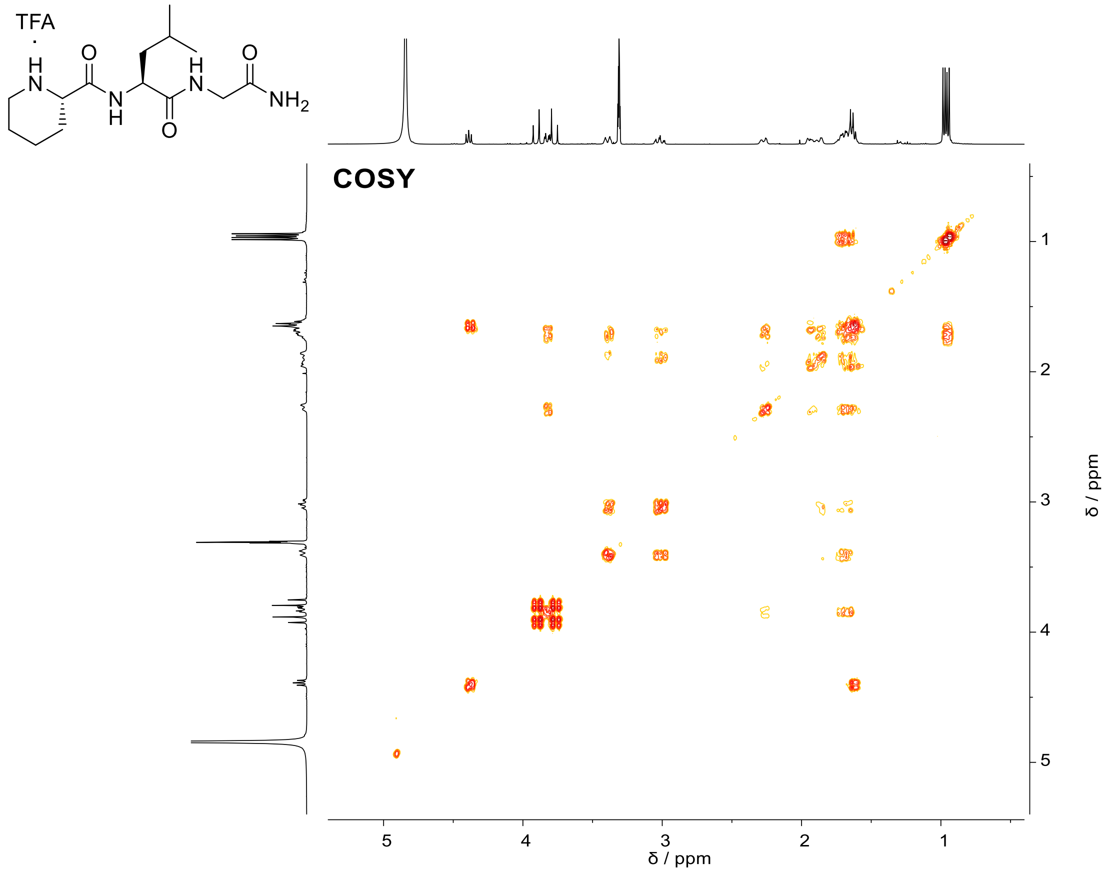

Figure S19. ^{1}H-^{1}H NMR (COSY) spectrum (CD_3OD, 400 MHz) of **1**.

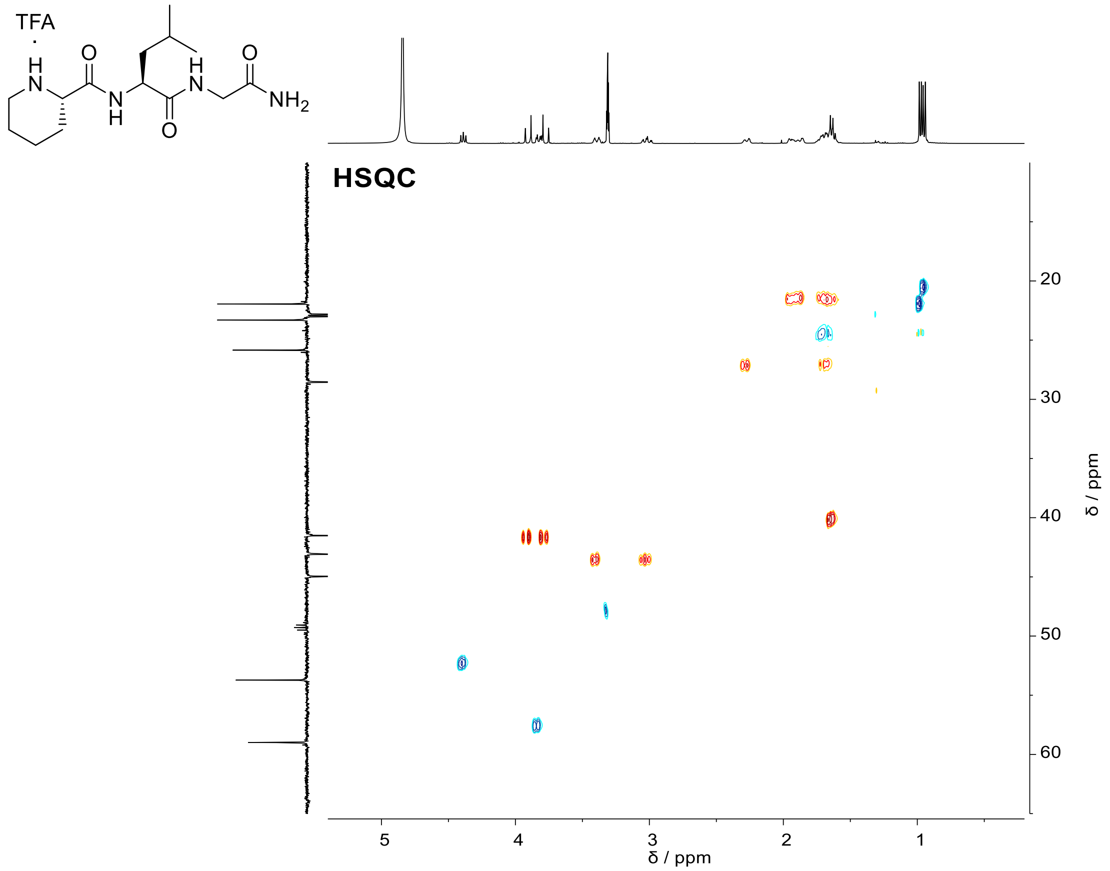

Figure S20. ^{1}H-^{13}C NMR (HSQC) spectrum (CD_3OD, 400 MHz) of **1**.

6. HRMS Spectra for Compounds 1, 7-9

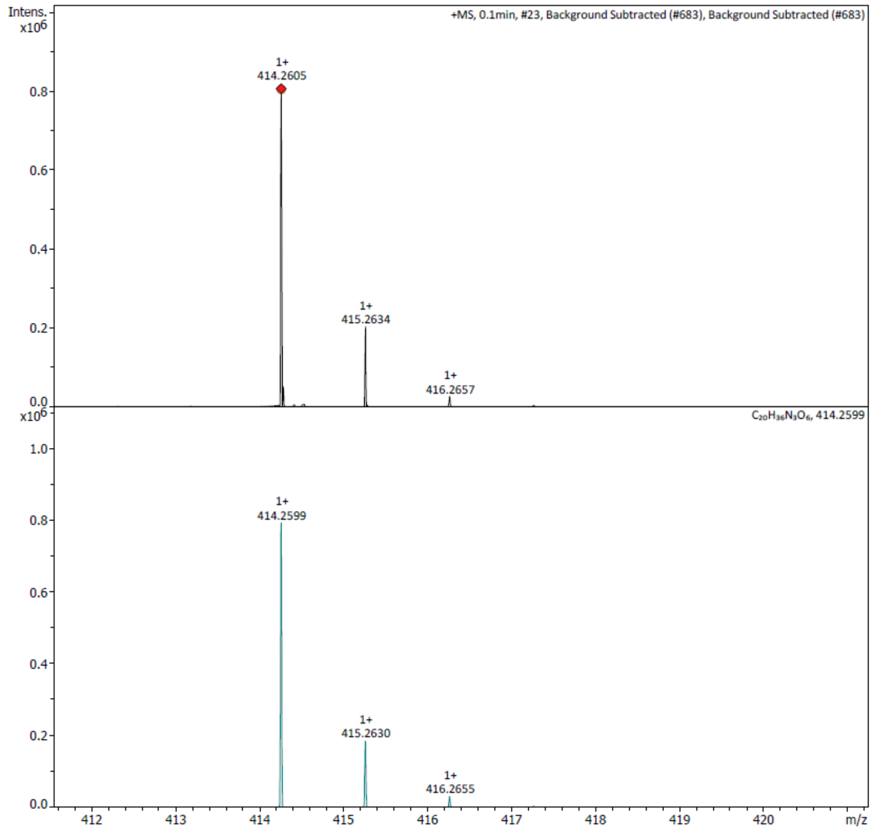

Figure S21. HRMS (ESI-TOF) spectrum of **7**, *m/z* calculated for $C_{20}H_{36}N_3O_6^+$: 414.2599; found: 414.2605.

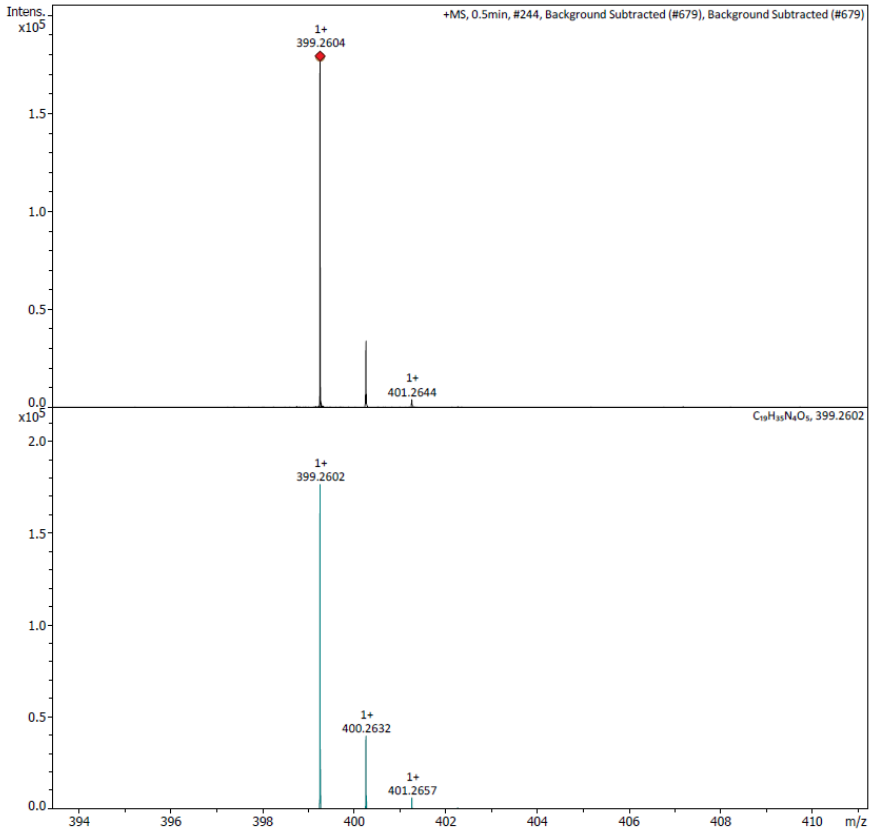

Figure S22. HRMS (ESI-TOF) spectrum of **8**, *m/z* calculated for $C_{19}H_{35}N_4O_5^+$: 399.2602; found: 399.2604.

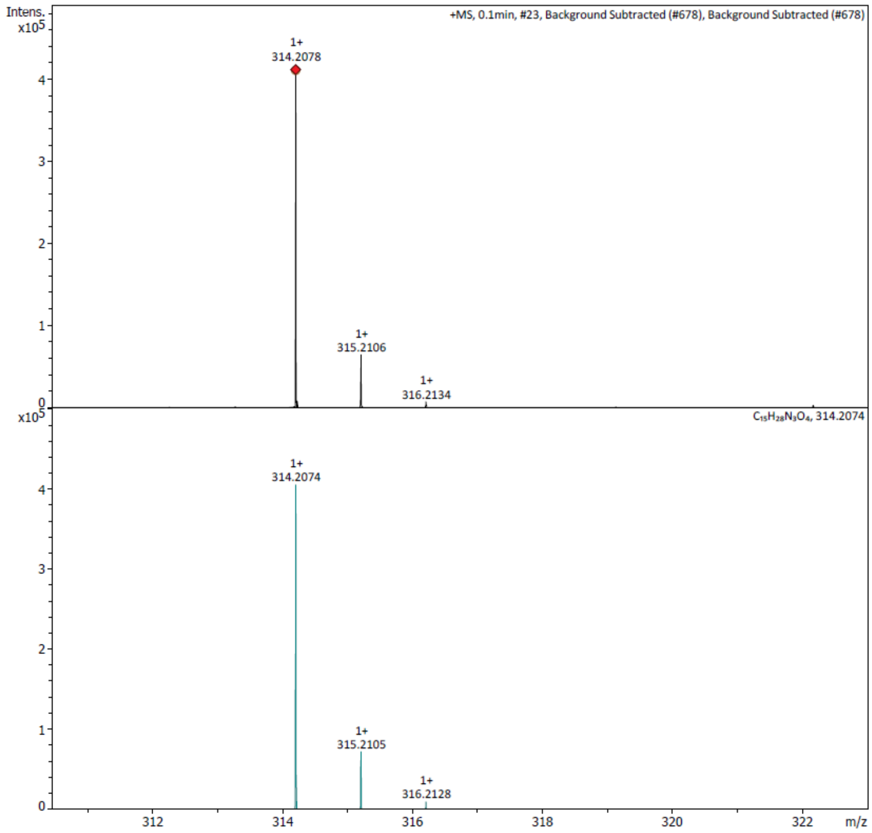

Figure S23. HRMS (ESI-TOF) spectrum of **9**, *m/z* calculated for $C_{15}H_{28}N_3O_4^+$: 314.2074; found: 314.2078.

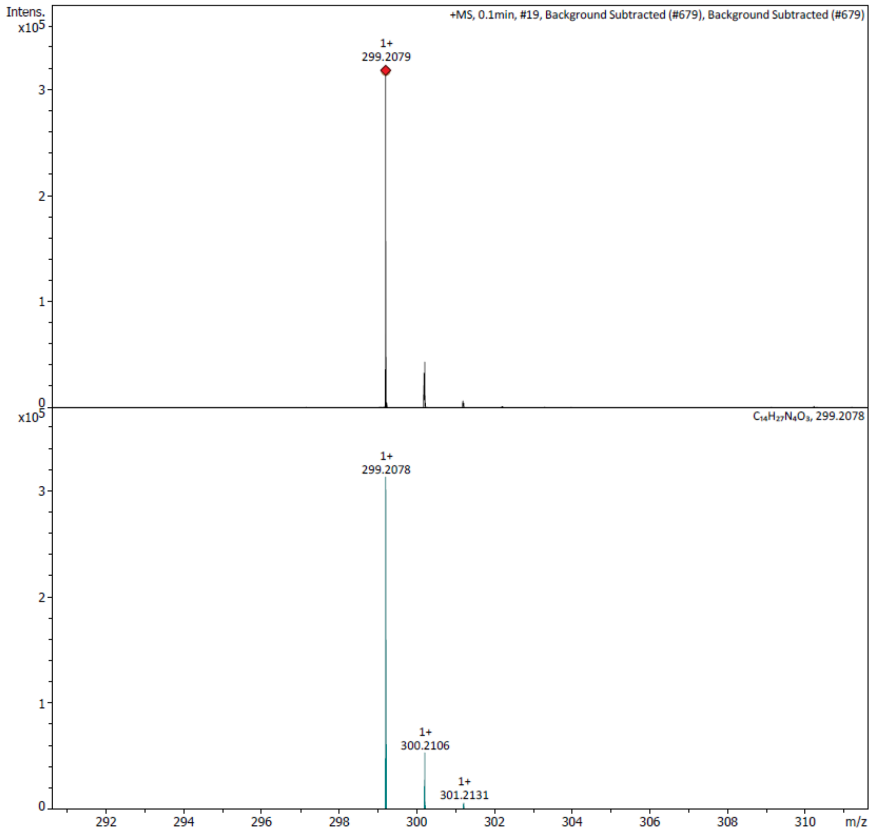

Figure S24. HRMS (ESI-TOF) spectrum of **1**, *m/z* calculated for $C_{14}H_{27}N_4O_3^+$: 299.2078; found: 299.2079.

7. Concentration-response curves of Dopamine in the presence of MIF-1

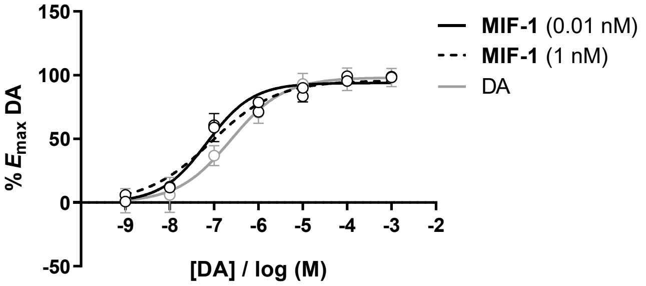

Figure S25. Concentration-response curves of dopamine (DA) in the presence of 0.01 nM (solid black line) and 1 nM (dashed black line) of MIF-1 (concentration-response curve of DA included as a gray solid line). Data points represent the mean and vertical bars represent the standard deviation of two independent experiments with duplicate measurements.

8. Conformational Analysis and Cartesian Coordinate Tables of Compounds 1 and 9

Compound **1** (Figure 4A) establishes an intramolecular hydrogen bond between the carbonyl oxygen of the L-pipecolyl residue $(C{=}O)_i$ and the *trans*-amide proton of the *C*-terminus $(NH)_{i+3}$, both in the neutral and protonated species. The hydrogen bond in the neutral species of **1** exhibits a donor-acceptor distance (D···A) of 2.95 Å and a hydrogen-acceptor distance (H···A) of 1.94 Å, with an associated bond angle (∠D–H···A) of 169.07°, while the protonated species of **1** displays D···A distance of 3.06 Å and an H···A distance of 2.06 Å, with ∠D–H···A of 169.00°, suggesting moderate-to-strong interactions for both species. These types of 10-membered pseudocyclic intraturns assisted by a hydrogen bond are collectively known as β-turns.[8, 9] The characteristic (φ, ψ) dihedral angles of the central residues (i + 1) and (i + 2) vary depending on their type.[8] The dihedral angles (i + 1) and (i + 2) obtained for the neutral and protonated species of **1** were (55.42°, 38.96°) and (94.70°, −10.35°), and (57.47°, 36.81°) and (91.27°, −6.96°), respectively, which are compatible with a type I' (inverse type I) β-turn conformation (Figure 4A), which ideally exhibits the dihedral angles (60°, 30°) and (90°, 0°).[8]

Conversely, compound **9** (Figure 4B) establishes a 7-membered pseudocyclic intraturn assisted by a moderate hydrogen bond between the $(C{=}O)_i$ and $(NH)_{i+2}$, known as a γ-turn, both in the neutral and protonated species.[10, 11] The neutral species of **9** exhibits D···A distance of 2.79 Å and an H···A distance of 1.83 Å, with ∠D–H···A of 154.39°, while the protonated species of **9** displays D···A distance of 2.84 Å and an H···A distance of 1.91 Å, with ∠D–H···A of 150.57°, suggesting moderate-to-strong interactions for both species.

According to Némethy and Printz,[12] based on the dihedral angles (φ, ψ) of the amino acid residue (i + 1), it is possible to categorize γ-turns into two types, namely classical and inverse γ-turns.[12] The classical γ-turn exhibits (φ, ψ) dihedral angles of approximately (75º, −64º), whereas the inverse γ-turn is defined by angles of (−79º, 69º).[12]

The (φ, ψ) angles obtained for compound **9** in the neutral and protonated states were found to be (71.78°, −57.98°) and (74.50°, −60.81°), respectively, which agree with classical γ-turn conformations (Figure 4B).[12]

Table S1. Cartesian coordinates of **1** (neutral), at the PBE0/6-31G(d,p) level of theory.

Atom	Cartesian coordinates			Atom	Cartesian coordinates		
	x	*y*	*z*		*x*	*y*	*z*
**C**	-0.9217	-7.0115	8.7499	**H**	-0.5169	-5.6533	5.7694
**O**	0.0250	-7.7830	8.9358	**H**	0.8114	-4.5488	7.7342
**N**	-2.7715	-5.6390	9.5988	**H**	1.8190	-5.9945	7.6751
**C**	-2.0705	-6.9095	9.7414	**H**	2.1926	-5.4877	5.1947
**C**	0.3719	-7.5468	6.0886	**H**	1.8318	-3.1413	4.4436
**O**	1.5249	-7.8109	5.7688	**H**	0.2809	-3.8766	4.8439
**N**	-1.0626	-6.2675	7.6378	**H**	1.0437	-2.7554	5.9808
**C**	-0.0358	-6.1608	6.6140	**H**	3.9367	-3.7594	5.6879
**C**	1.1777	-5.3440	7.0723	**H**	3.9143	-5.0385	6.9134
**C**	1.9938	-4.7079	5.9395	**H**	3.1875	-3.4620	7.2628
**C**	1.2419	-3.5621	5.2646	**H**	-1.5701	-8.1473	6.2401
**C**	3.3347	-4.2165	6.4802	**H**	-1.2892	-10.0519	4.8082
**C**	-0.2468	-10.8626	6.4696	**H**	0.4499	-9.7469	4.8062
**O**	-0.2470	-12.0342	6.0989	**H**	-0.0236	-9.5042	8.0347
**N**	-0.6471	-8.4268	5.9441	**H**	0.1075	-11.1950	8.4284
**C**	-0.4448	-9.7562	5.4356	**H**	-4.3759	-4.5361	10.3317
**N**	-0.0646	-10.4769	7.7423	**H**	-3.6113	-5.5594	11.5577
**C**	-3.9209	-5.5177	10.4980	**H**	-5.7691	-6.5264	10.9312
**C**	-4.9265	-6.6288	10.2392	**H**	-5.3234	-6.5263	9.2211
**C**	-4.2555	-7.9899	10.3948	**H**	-4.9548	-8.8002	10.1626
**C**	-3.0356	-8.0769	9.4844	**H**	-3.9433	-8.1264	11.4392
**H**	-2.1233	-4.8859	9.8135	**H**	-3.3628	-8.0306	8.4371
**H**	-1.6236	-7.0442	10.7415	**H**	-2.4960	-9.0191	9.6213
**H**	-1.8610	-5.6412	7.6332				

Table S2. Cartesian coordinates of **1** (protonated), at the PBE0/6-31G(d,p) level of theory.

Atom	Cartesian coordinates			Atom	Cartesian coordinates		
	x	*y*	*z*		*x*	*y*	*z*
**C**	0.9260	-0.1392	0.0138	**H**	0.5682	-1.2066	-1.6754
**O**	0.5054	0.3999	1.0406	**H**	-1.6714	-1.3216	-1.6222
**N**	3.0847	0.2766	0.9994	**H**	-1.4663	-1.5119	1.3997
**C**	2.4230	-0.0662	-0.2971	**H**	-0.7562	-2.7534	0.3665
**C**	-2.0190	0.3667	-0.3917	**H**	-3.6823	-1.9406	0.2195
**O**	-2.9698	0.4298	0.3766	**H**	-2.2212	-4.4297	-0.8186
**N**	0.1427	-0.7401	-0.8879	**H**	-2.8239	-3.1154	-1.8396
**C**	-1.2859	-0.9568	-0.6638	**H**	-3.9598	-4.1259	-0.9499
**C**	-1.5535	-2.0015	0.4251	**H**	-2.4074	-4.2550	1.7655
**C**	-2.9094	-2.7117	0.3189	**H**	-4.1462	-4.0034	1.5577
**C**	-2.9799	-3.6411	-0.8915	**H**	-3.1861	-2.8322	2.4771
**C**	-3.1790	-3.4923	1.6034	**H**	-0.8013	1.3146	-1.7325
**C**	-1.6596	3.6801	0.0484	**H**	-2.1547	3.2325	-1.9675
**O**	-2.0855	4.8309	0.0912	**H**	-3.2701	2.5725	-0.7685
**N**	-1.5773	1.4328	-1.0989	**H**	-0.3805	3.7943	1.6115
**C**	-2.2123	2.7212	-1.0039	**H**	-0.4191	2.2274	0.8593
**N**	-0.7315	3.1898	0.8855	**H**	4.9187	0.8266	1.8479
**C**	4.5450	0.5743	0.8547	**H**	4.6208	1.4560	0.2139
**C**	5.2454	-0.6315	0.2552	**H**	5.2296	-1.4580	0.9765
**C**	4.5978	-1.0605	-1.0591	**H**	6.2956	-0.3694	0.1009
**C**	3.0940	-1.2943	-0.8980	**H**	5.0686	-1.9759	-1.4270
**H**	2.9601	-0.5035	1.6525	**H**	4.7629	-0.2877	-1.8196
**H**	2.5626	1.0529	1.4210	**H**	2.6525	-1.5024	-1.8767
**H**	2.5791	0.8016	-0.9499	**H**	2.9000	-2.1672	-0.2631

Table S3. Cartesian coordinates of **9** (neutral), at the PBE0/6-31G(d,p) level of theory.

Atom	Cartesian coordinates			Atom	Cartesian coordinates		
	x	*y*	*z*		*x*	*y*	*z*
**C**	-4.8173	0.9473	-0.2594	**H**	-1.6177	0.0416	-0.1894
**O**	-5.3168	0.9982	0.8730	**H**	-1.9585	2.2561	0.7754
**N**	-4.8182	1.5871	-2.6336	**H**	-3.1502	1.6328	1.9125
**C**	-5.6593	1.2633	-1.4881	**H**	-1.4170	0.2740	3.0360
**C**	-2.7394	-1.1056	1.1490	**H**	1.0087	0.6616	2.4976
**O**	-1.8888	-1.9928	1.1243	**H**	0.1569	-0.1879	1.2053
**N**	-3.5633	0.5547	-0.5326	**H**	0.5286	1.5416	1.0396
**C**	-2.5062	0.2245	0.4175	**H**	-0.2063	2.1604	4.0897
**C**	-2.2238	1.3826	1.3858	**H**	-1.8994	2.5961	3.8055
**C**	-1.1104	1.1215	2.4058	**H**	-0.6326	3.2158	2.7349
**C**	0.2191	0.7611	1.7455	**H**	-4.5672	-0.4328	1.7356
**C**	-0.9555	2.3410	3.3121	**H**	-3.3475	-2.9704	2.7274
**C**	-5.2173	-3.3202	1.8093	**H**	-4.7114	-2.1644	3.5036
**O**	-5.6880	-4.3076	2.3311	**H**	-6.4852	-3.3299	-1.1460
**N**	-3.8923	-1.1930	1.8455	**H**	-5.9319	-4.7928	-0.2732
**C**	-4.2580	-2.3946	2.5350	**H**	-7.3230	-3.8680	0.3433
**O**	-5.4714	-2.9408	0.5582	**H**	-4.8748	2.0792	-4.6536
**C**	-6.3616	-3.7941	-0.1690	**H**	-6.2618	2.6976	-3.7437
**C**	-5.5839	1.8325	-3.8569	**H**	-6.9817	0.8181	-5.1379
**C**	-6.4079	0.6097	-4.2287	**H**	-5.7317	-0.2256	-4.4507
**C**	-7.3323	0.2307	-3.0756	**H**	-7.8946	-0.6802	-3.3081
**C**	-6.5238	0.0322	-1.7983	**H**	-8.0705	1.0298	-2.9214
**H**	-4.2866	2.4265	-2.4189	**H**	-5.8575	-0.8326	-1.9167
**H**	-6.3269	2.0925	-1.1971	**H**	-7.1713	-0.1666	-0.9388
**H**	-3.3489	0.5395	-1.5252				

Table S4. Cartesian coordinates of **9** (protonated), at the PBE0/6-31G(d,p) level of theory.

Atom	Cartesian coordinates			Atom	Cartesian coordinates		
	x	*y*	*z*		*x*	*y*	*z*
**C**	1.0997	-0.4258	-0.0358	**H**	0.5821	-0.7871	-1.9454
**O**	0.8222	-0.2496	1.1558	**H**	-1.5167	-1.4156	-1.8066
**N**	3.2252	0.5458	0.5530	**H**	-0.9331	-1.9728	1.1371
**C**	2.5631	-0.3291	-0.4637	**H**	-0.7806	-3.1017	-0.2066
**C**	-2.0678	0.1164	-0.4985	**H**	-3.3753	-1.8407	0.7984
**O**	-3.0180	0.3994	-1.2222	**H**	-2.9387	-3.9020	-1.4252
**N**	0.2320	-0.7453	-0.9989	**H**	-3.5991	-2.2639	-1.6169
**C**	-1.1807	-1.0968	-0.8191	**H**	-4.5096	-3.4855	-0.7259
**C**	-1.3658	-2.2533	0.1718	**H**	-2.3323	-4.7078	1.0260
**C**	-2.8203	-2.6918	0.3781	**H**	-3.8981	-4.1236	1.6078
**C**	-3.5024	-3.1053	-0.9246	**H**	-2.4016	-3.5310	2.3475
**C**	-2.8654	-3.8257	1.4003	**H**	-0.9167	0.5390	1.1292
**C**	-1.9524	3.2825	0.4589	**H**	5.0590	1.2602	1.2563
**O**	-2.4519	4.3523	0.7301	**H**	4.9174	1.1547	-0.5098
**N**	-1.7683	0.7886	0.6330	**H**	5.1981	-1.2051	1.4332
**C**	-2.4911	1.9707	1.0029	**H**	6.4148	-0.6306	0.2996
**O**	-0.8951	3.1262	-0.3345	**H**	5.1531	-2.6374	-0.5882
**C**	-0.3592	4.3341	-0.8855	**H**	4.9738	-1.2275	-1.6286
**C**	4.7177	0.6346	0.4303	**H**	2.7597	-2.2924	-1.2791
**C**	5.3374	-0.7476	0.4460	**H**	2.9671	-2.2223	0.4686
**C**	4.7294	-1.6307	-0.6381	**H**	-2.5286	2.0649	2.0915
**C**	3.2152	-1.7133	-0.4713	**H**	-3.5210	1.8820	0.6457
**H**	2.9365	0.1955	1.4757	**H**	-0.0342	5.0058	-0.0883
**H**	2.8268	1.4864	0.4982	**H**	0.4889	4.0296	-1.4965
**H**	2.6747	0.1663	-1.4328	**H**	-1.1090	4.8387	-1.4983

9. References

1. Gottlieb, H. E.; Kotlyar, V.; Nudelman, A., NMR chemical shifts of common laboratory solvents as trace impurities. *J. Org. Chem.* **1997**, *62* (21), 7512-7515.
2. Silva-Reis, S. C.; Correia, X. C.; Costa-Almeida, H. F.; Pires-Lima, B. L.; Maronde, D.; Costa, V. M.; García-Mera, X.; Cruz, L.; Brea, J.; Loza, M. I.; Rodríguez-Borges, J. E.; Sampaio-Dias, I. E., Stapling Amantadine to Melanostatin neuropeptide: Discovery of potent positive allosteric modulators of the D_2 receptors. *ACS Med. Chem. Lett.* **2023**, *14* (12), 1656-1663.
3. Sampaio-Dias, I. E.; Reis-Mendes, A.; Costa, V. M.; García-Mera, X.; Brea, J.; Loza, M. I.; Pires-Lima, B. L.; Alcoholado, C.; Algarra, M.; Rodríguez-Borges, J. E., Discovery of new potent positive allosteric modulators of dopamine D_2 receptors: Insights into the bioisosteric replacement of proline to 3-furoic acid in the Melanostatin neuropeptide. *J. Med. Chem.* **2021**, *64* (9), 6209-6220.
4. Sampaio-Dias, I. E.; Silva-Reis, S. C.; García-Mera, X.; Brea, J.; Loza, M. I.; Alves, C. S.; Algarra, M.; Rodríguez-Borges, J. E., Synthesis, pharmacological, and biological evaluation of MIF-1 picolinoyl peptidomimetics as positive allosteric modulators of D_2R. *ACS Chem. Neurosci.* **2019**, *10* (8), 3690-3702.
5. Silva-Reis, S. C.; Costa, V. M.; Correia da Silva, D.; Pereira, D. M.; Correia, X. C.; Costa-Almeida, H. F.; García-Mera, X.; Rodríguez-Borges, J. E.; Sampaio-Dias, I. E., Exploring structural determinants of neuroprotection bias on novel Glypromate conjugates with bioactive amines. *Eur. J. Med. Chem.* **2024**, *267*, 116174.
6. Fotakis, G.; Timbrell, J. A., *In vitro* cytotoxicity assays: Comparison of LDH, neutral red, MTT and protein assay in hepatoma cell lines following exposure to cadmium chloride. *Toxicol. Lett.* **2006**, *160* (2), 171-177.
7. Repetto, G.; del Peso, A.; Zurita, J. L., Neutral red uptake assay for the estimation of cell viability/cytotoxicity. *Nat. Protoc.* **2008**, *3* (7), 1125-1131.
8. Wilmot, C. M.; Thornton, J. M., Analysis and prediction of the different types of β-turn in proteins. *J. Mol. Biol.* **1988**, *203* (1), 221-232.
9. Gao, W.; Han, J.; Greaves, S.; Harrity, J. P. A., Asymmetric synthesis of functionalizable type II β-turn-inducing α-amino acid building blocks. *Org. Lett.* **2023**, *25* (35), 6555-6559.
10. Chou, K. C., Prediction of tight turns and their types in proteins. *Anal. Biochem.* **2000**, *286* (1), 1-16.
11. Rosenstrom, U.; Skold, C.; Plouffe, B.; Beaudry, H.; Lindeberg, G.; Botros, M.; Nyberg, F.; Wolf, G.; Karlen, A.; Gallo-Payet, N.; Hallberg, A., New selective AT2 receptor ligands encompassing a gamma-turn mimetic replacing the amino acid residues 4-5 of angiotensin II act as agonists. *J. Med. Chem.* **2005**, *48* (12), 4009-4024.
12. Némethy, G.; Printz, M. P., The γ turn, a possible folded conformation of the polypeptide chain. Comparison with the β turn. *Macromolecules* **1972**, *5* (6), 755-758.
